# NLR-Associating Transcription Factor bHLH84 and Its Paralogs Function Redundantly in Plant Immunity

**DOI:** 10.1371/journal.ppat.1004312

**Published:** 2014-08-21

**Authors:** Fang Xu, Paul Kapos, Yu Ti Cheng, Meng Li, Yuelin Zhang, Xin Li

**Affiliations:** 1 Michael Smith Laboratories, University of British Columbia, Vancouver, British Columbia, Canada; 2 Department of Botany, University of British Columbia, Vancouver, British Columbia, Canada; 3 National Institute of Biological Sciences, Beijing, People's Republic of China; Wageningen University, Netherlands

## Abstract

In plants and animals, nucleotide-binding and leucine-rich repeat domain containing (NLR) immune receptors are utilized to detect the presence or activities of pathogen-derived molecules. However, the mechanisms by which NLR proteins induce defense responses remain unclear. Here, we report the characterization of one basic Helix-loop-Helix (bHLH) type transcription factor (TF), bHLH84, identified from a reverse genetic screen. It functions as a transcriptional activator that enhances the autoimmunity of *NLR* mutant *snc1* (*suppressor of npr1-1, constitutive 1*) and confers enhanced immunity in wild-type backgrounds when overexpressed. Simultaneously knocking out three closely related *bHLH* paralogs attenuates RPS4-mediated immunity and partially suppresses the autoimmune phenotypes of *snc1*, while overexpression of the other two close paralogs also renders strong autoimmunity, suggesting functional redundancy in the gene family. Intriguingly, the autoimmunity conferred by *bHLH84* overexpression can be largely suppressed by the loss-of-function *snc1-r1* mutation, suggesting that *SNC1* is required for its proper function. *In planta* co-immunoprecipitation revealed interactions between not only bHLH84 and SNC1, but also bHLH84 and RPS4, indicating that bHLH84 associates with these NLRs. Together with previous finding that SNC1 associates with repressor TPR1 to repress negative regulators, we hypothesize that nuclear NLR proteins may interact with both transcriptional repressors and activators during immune responses, enabling potentially faster and more robust transcriptional reprogramming upon pathogen recognition.

## Author Summary

In plants and animals, NLR immune receptors are utilized to detect pathogen-derived molecules and activate immunity. However, the mechanisms of plant NLR activation remain unclear. Here, we report on bHLH84, which functions as a transcriptional activator. Simultaneously knocking out three closely related *bHLH* paralogs partially suppresses the autoimmunity of *snc1* and compromises RPS4-mediated defense, while overexpression of these close paralogs renders strong autoimmunity, suggesting functional redundancy in the gene family. *In planta* co-immunoprecipitation revealed interactions between not only bHLH84 and SNC1, but also bHLH84 and RPS4. Therefore bHLH84 family transcription factors associate with these NLRs to activate defense responses, enabling potentially faster and more robust transcriptional reprogramming upon pathogen recognition.

## Introduction

Plants have evolved a sophisticated immune system to fight against invading microbial pathogens that threaten their normal growth and development. Plant immunity is in part mediated by resistance (R) proteins that recognize pathogen proteins known as effectors [1]–[3]. The majority of R proteins are NLR receptors that contain leucine-rich repeats (LRRs) at the C-terminus, a central nucleotide-binding site (NBS) and either a Toll/Interleukin-1 receptor (TIR) or a coiled-coil (CC) domain at the N-terminus [4]. In Arabidopsis, genetically downstream of the R proteins are the EDS1 (ENHANCED DISEASE SUSCEPTIBILITY 1)/PAD4 (PHYTOALEXIN DEFICIENT 4)/SAG101 (SENESCENCE-ASSOCIATED GENE101) complex and NDR1 (NON-RACE-RESISTANCE 1), which mainly mediate TIR-NB-LRR or CC-NB-LRR triggered defense responses, respectively [5]–[8].

While the mechanisms underlying effector recognition by R proteins have been intensively studied, little is known about the post-recognition events leading to defense activation. Recently, it has been shown that the nuclear pool of certain R proteins, including MLA10 (MILDEW A LOCUS 10) in barley, N in tobacco, Pb1 (Panicle blast 1) in rice, and RPS4 (RESISTANT TO *P.SYRINGAE* 4), RRS1 (RESISTANT TO *RALSTONIA SOLANACEARUM* 1) and SNC1 (SUPPRESSOR OF *NPR1-1*, CONSTITUTIVE1) in Arabidopsis, is important for the activation of defense responses [9]–[14]. The latest discoveries on the interactions between some of these R proteins and their associating transcription factors (TFs) further shed light on the activation mechanism of nuclear R proteins. For example, MLA10 interacts with WRKY TFs to de-repress PAMP (PATHOGEN-ASSOCIATED MOLECULAR PATTERN) triggered basal defense [9]. The active state of MLA10 can also release MYB6 (MYB DOMAIN PROTEIN 6) from WRKY suppression and promote its binding to cis-elements to initiate defense responses [15]. CC-type NLR Pb1 in rice interacts with WRKY45 and this interaction is believed to protect the TF from proteasomal degradation in the nucleus [16]. In addition, SNC1 associates with transcriptional co-repressor TPR1 (TOPLESS RELATED 1) to negatively regulate the expression of known defense suppressors, thereby activating plant immunity [17]. Lately, studies on N in tobacco showed that it is able to associate with the TF SPL6 (SQUAMOSA PROMOTER BINDING PROTEIN-LIKE 6) upon effector recognition [18]. From these data, it has been hypothesized that some NLRs associate with TFs inside the nucleus to directly participate in transcriptional reprogramming to regulate downstream defense responses.

In Arabidopsis, the gain-of-function *NLR* mutant *snc1* constitutively expresses *PATHOGENESIS RELATED* (*PR*) defense marker genes and exhibits enhanced disease resistance against virulent bacteria *Pseudomonas syringae* pv. *maculicola* (*P.s.m.*) ES4326 and oomycete *Hyaloperonospora arabidopsidis* (*H.a.*) Noco2 [19], [20]. As *snc1* displays strong autoimmune phenotypes while remaining fully fertile, it has become a useful tool for dissecting NLR mediated resistance. Forward genetic screens designed to isolate positive regulators of immunity were conducted in the *snc1* background and over a dozen *Modifier of snc1* (*MOS*) genes have been identified. Characterizations of the *MOS* genes and their encoded protein products have revealed complicated regulatory events surrounding *snc1* mediated autoimmunity, which include nucleocytoplasmic trafficking, RNA processing, protein modification and transcriptional regulation [21], [22]. However, genetic redundancy and lethality may have prevented some essential positive regulators from being discovered through forward genetic approaches. Here, we employed a targeted reverse genetic screen to search for candidate TFs participating in the regulation of *snc1*-mediated defense. One basic Helix-loop-Helix (bHLH) type TF, which is a putative transcriptional activator, was isolated from the screen and found to be able to associate with NLRs to activate immunity.

## Results

### A targeted reverse genetic screen

Previously, SNC1 was found to participate directly in transcriptional reprogramming with TPR/MOS10 repressor proteins that do not directly bind DNA [17]. We did not find a DNA-binding TF that functions together with SNC1 from the MOS forward genetic screens, suggesting that multiple TFs may function redundantly in *snc1*-mediated immunity. To search for novel TFs regulating plant immunity, a reverse genetic screen was employed. As UV irradiation has been shown to induce resistance to pathogens and to induce transcription of defense related genes [23]–[25], we selected 36 putative TFs which show >1.7-fold enhanced expression level upon UV treatment based on publically available microarray data from The Arabidopsis Information Resource (Table S1). The genomic sequences of these genes were cloned into a binary vector *pCambia1305* containing C-terminus *GFP* and *HA* double tags. Using the floral dip method [26], overexpression transgenic plants in *snc1* and Col-0 backgrounds were generated. From the primary screen, we searched for transformants either suppressing or enhancing the dwarf morphology of *snc1* or causing dwarfism in Col-0 background. Transgenic plants exhibiting heritable altered morphology were subject to a secondary screen, where altered resistance was examined using a *Hyaloperonospora arabidopsidis* (*H.a.*) Noco2 infection assay. Screening data for these candidate TFs are summarized in Table S1.

**Figure 1:**
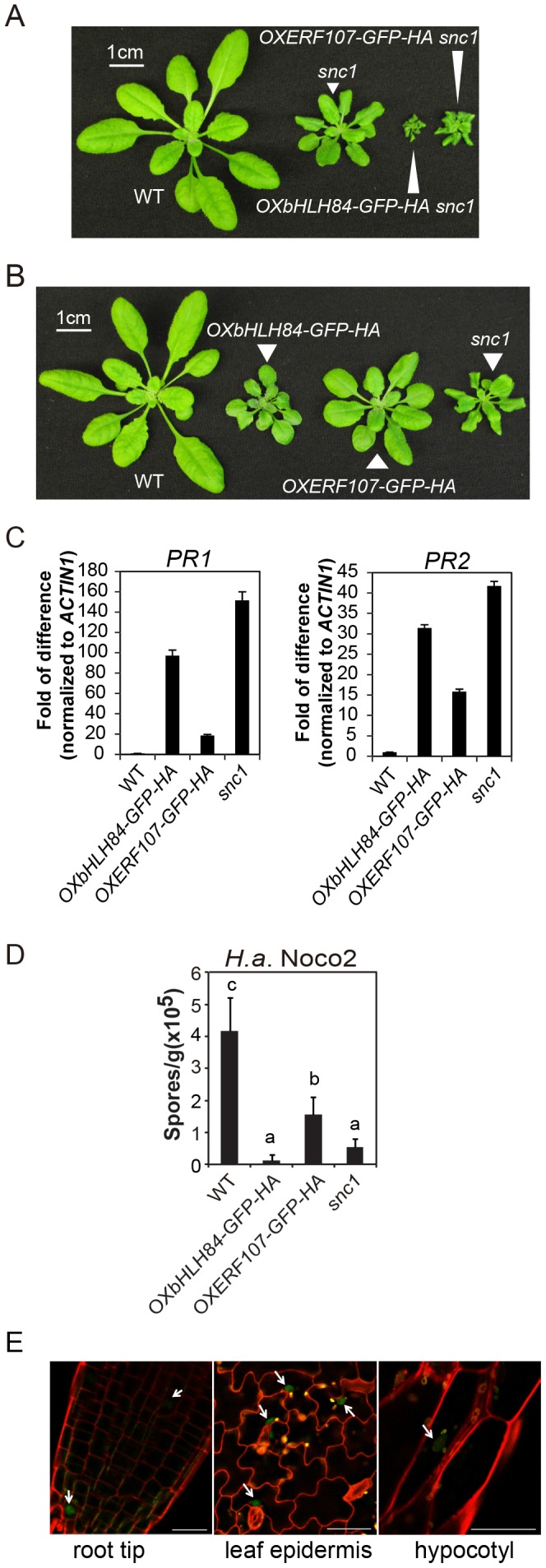
Characterization of *bHLH84* and *ERF107* overexpression (OX) lines. A. Morphology of wild type (WT), *snc1* and representative transgenic lines of *OXbHLH84-GFP-HA* and *OXERF107-GFP-HA* in *snc1* background. For both A and B, plants were grown on soil for four weeks before the pictures were taken. B. Morphology of WT, *OXbHLH84-GFP-HA*, *OXERF107-GFP-HA* and *snc1* plants. Genes were overexpressed in Col-0 WT background. C. Relative *PR1* and *PR2* gene expression in WT, *OXbHLH84-GFP-HA*, and *OXERF107-GFP-HA* plants as determined by real-time PCR. Total RNA samples were extracted from 12-day-old plants grown on solid MS medium and reverse transcribed to cDNA using Superscript II reverse transcriptase. All genotypes of plants were grown simultaneously on the same large petri plate. The expression of *PR1* and *PR2* was normalized to that of *ACTIN1*, and the value of each genotype was compared to that of WT. D. Quantification of *H.a.* Noco2 sporulation on WT, *OXbHLH84-GFP-HA*, *OXERF107-GFP-HA* and *snc1* plants. 2.5-week-old plants were inoculated with *H.a.* Noco2 at a concentration of 10^5^ spores/mL water. Oomycete spores on the leaf surface were quantified seven days after inoculation. Bars represent means of four replicates ± SD. Variant letters represent statistical differences among the indicated genotypes as analyzed by StatsDirect software (*p*<0.05). E. Detection of GFP green fluorescence in *OXbHLH84-GFP-HA* seedlings using confocal fluorescence microscopy. The pictures were taken from root tip and leaf epidermis of 10-day-old plants and hypocotyl of 5-day-old seedlings. Cell walls were visualized in red using propidium iodide (PI) staining. Arrows point to the nuclei with GFP signal. Scale bar = 20 µm.

From the screen, we identified several TFs that displayed phenotypes in only *snc1* or Col-0 background, but not in both when overexpressed (Table S1). However, overexpression of three TFs, *At2g31230*, *At2g14760* or *At5g61590*, resulted in stunted growth in both the *snc1* and Col-0 backgrounds (Table S1, Figure 1A and 1B). We selected two TFs with the strongest phenotypes for further analysis. *At2g14760* encodes bHLH84, a predicted basic helix-loop-helix TF, while *At5g61590* encodes ERF107, which belongs to the ethylene-response-factor (ERF) TF family.

### Characterization of the *OXbHLH84-GFP-HA* and *OXERF107-GFP-HA* lines

To further explore the functions of *bHLH84* and *ERF107* in plant immunity, we isolated homozygous overexpression transgenic lines in Col-0 background. As shown in Figure 1B, both *OXbHLH84-GFP-HA* and *OXERF107-GFP-HA* plants exhibited dwarf morphology compared with WT plants. We further examined defense marker *PR* gene expression in these transgenic plants using real-time PCR. As shown in Figure 1C, the expression of both *PR1* and *PR2* was significantly up-regulated, with about 100- and 35- fold changes, respectively, in *OXbHLH84-GFP-HA*, indicating that the defense responses were constitutively activated. In *OXERF107-GFP-HA* transgenic plants, both *PR1* and *PR2* were around 15-fold up-regulated. Consistent with *PR* gene expression, resistance against virulent pathogen *H.a.* Noco2 was enhanced in both *OXbHLH84-GFP-HA* and *OXERF107-GFP-HA* plants (Figure 1D). As *OXbHLH84-GFP-HA* plants displayed more severe immune phenotypes than *OXERF107-GFP-HA* plants, we chose to focus solely on the functional study of *bHLH84*. Consistent with its predicted TF function, bHLH84-GFP-HA fluorescence was detected in the nuclei when the *OXbHLH84-GFP-HA* seedlings were examined by confocal fluorescence microscopy (Figure 1E).

### bHLH84 functions as a transcriptional activator

**Figure 2:**
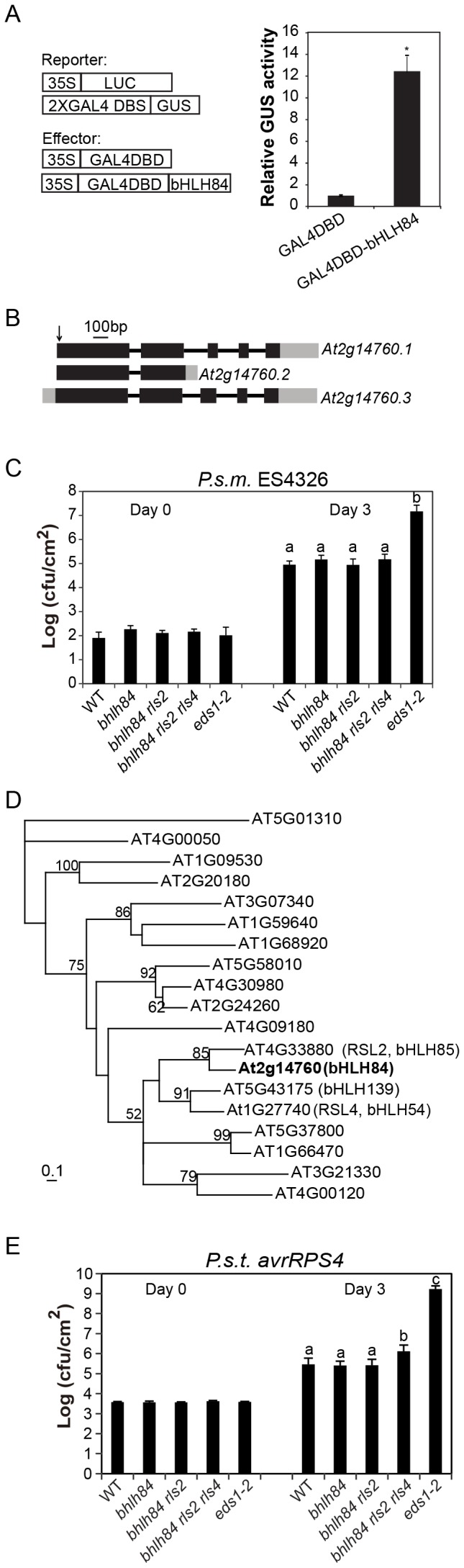
Mutant analysis of transcriptional activator *bHLH84* and its paralogs. A. Relative GUS activities were assayed using Arabidopsis mesophyll protoplasts cotransfected with the reporter and the indicated effector constructs. The 35S-driven luciferase (LUC) report construct served as internal transfection control. * indicates statistical significance analyzed by unpaired student's t-test (*p*<0.01). B. Gene structure of *bHLH84* (*At2g14760*). There are three different splice variants of *bHLH84*. Boxes indicate exons and lines indicate introns. Grey regions show the UTR regions. The position of the T-DNA insertion in *bhlh84* is indicated with an arrow. C. Bacterial growth of *P.s.m.* ES4326 on four-week-old plants of the indicated genotypes at 0 (Day 0) and 3 days (Day 3) post-inoculation, with bacterial inoculum of OD_600_ = 0.0001. *eds1-2* plants served as enhanced disease susceptibility (EDS) control. Bars represent means of five replicates ±SD. Statistical difference among the indicated genotypes were analyzed with a StatsDirect software (*p*<0.001). D. A phylogenetic tree of bHLH84 and its close paralogs in Arabidopsis. The amino acid sequences of bHLH84 and its paralogs in Arabidopsis were used to generate the tree, using a method as previously described [61]. E. Bacterial growth of the avirulent pathogen *P.s.t. avrRPS4* on plants of the indicated genotype. Four-week-old plants were infiltrated with a bacterial suspension at OD_600_ = 0.002. Leaf discs within the infected area were taken at day 0 and day 3 to measure the bacterial growth. Bars represent means of five replicates ±SD. Statistical differences were analyzed by one-way analysis using StatsDirect software. Variant letters represent statistical difference among the indicated genotype. (*p*<0.05). For each experiment, five plants with two leaves per plant were inoculated. Two leaf discs from each plant were assayed as one replicate. Similar results were observed in four independent trials.

To further investigate how bHLH84 regulates plant immunity, we tested whether it is a *bona fide* transcription factor by conducting a previously established protoplast transcription activity transient assay [27]. In this assay, the β-glucuronidase (GUS) reporter gene is driven by 2×Gal4 DNA-binding sites (DBS). Co-transformation of *bHLH84* fused with the Gal4 DNA-binding domain (DBD) together with the reporter constructs in Arabidopsis mesophyll protoplasts resulted in drastically enhanced GUS expression (Figure 2A) compared to the control transfection, suggesting that bHLH84 functions as a transcriptional activator.

### Knocking out *bHLH84* and its two close paralogs does not compromise basal immunity while attenuating RPS4-mediated defense response

bHLH TFs constitute one of the largest TF families in Arabidopsis, with 147 members including *bHLH84* [28]. *bHLH84* has three alternatively spliced variants according to available expressed sequence tag (EST) data (Figure 2B). Based on sequence analysis, *At2g14760.2* encodes a truncated protein without the C-terminal bHLH DNA binding domain, while the other two variants encode full-length proteins [28]. However, when the coding region of *bHLH84* was amplified from cDNA of WT plants and sequenced, only *At2g14760.1* was observed, suggesting that *At2g14760.1* is the dominantly expressed version.

To further investigate the contribution of *bHLH84* in plant immunity, knock-out analysis of *bHLH84* was carried out. A T-DNA allele of *bHLH84* (SALK_064296) was obtained from the Arabidopsis Biological Resource Centre (ABRC). As shown in Figure 2B, the T-DNA inserts in the first exon of *At2g14760.1*. As a consequence, the expression of *bHLH84* was abolished (Figure S1A). SALK_064296 was thus assigned as *bhlh84*. When *bhlh84* leaves were challenged with virulent bacterial pathogen *Pseudomonas syringae* pv *maculicola* (*P.s.m.*) ES4326, they exhibited similar bacterial growth as WT (Figure 2C), indicating that the immune response is not compromised in the knock-out mutant.

To investigate whether genetic redundancy masks the function of *bHLH84*, we carried out a phylogenetic analysis of bHLH84 and its paralogs. As *RSL2* (*ROOT HAIR DEFECTIVE 6-LIKE 2*) is the closest paralog of *bHLH84* (Figure 2D; [29]), a T-DNA knock-out line for this gene, SALK_048849, was obtained from ABRC. As shown in Figure S1B, no expression of *RSL2* was detectable in SALK_048849, which was named as *rsl2*. Double mutant *bhlh84 rsl2* was created and subjected to pathogen infection experiments. As shown in Figure 2C, the *bhlh84 rsl2* double mutant did not exhibit resistance defects either. As *RSL4* (*ROOT HAIR DEFECTIVE 6-LIKE 4*) is functionally redundant with *RSL2* in regulating root hair growth [29], we further created the triple mutant by crossing *bhlh84 rsl2* with *rsl4 rsl2*, which was characterized by Yi et al., 2010 [29]. The triple mutant *bhlh84 rsl2 rsl4* still did not exhibit obvious defects upon infection with *P.s.m.* ES4326 compared to WT plants (Figure 2C), indicating that knocking out *bHLH84* and its two paralogs does not compromise basal defense responses. Since no good T-DNA mutant line was available for *bHLH139*, we were not able to test higher level of redundancy using knockout approach.

To further examine the contribution of these TFs in specific R protein mediated immunity, we challenged single, double and triple mutant plants with *Pseudomonas syringae* pv *tomato* (*P.s.t.*) carrying either *avrRPS4* or *hopA1*, which are effectors recognized by TIR-NB-LRR proteins RPS4 and RPS6, respectively. As shown in Figure 2E, significantly more *P.s.t. avrRPS4* growth was observed in *bhlh84 rsl2rsl4* triple mutant plant, while no detectable difference was observed when the TF mutants were challenged with *P.s.t. hopA1* (Figure S2), suggesting that these bHLH TFs contribute redundantly to RPS4-mediated immunity.

### Simultaneously knocking out *bHLH84*, *RSL2* and *RSL4* partially suppresses the autoimmunity of *snc1*

**Figure 3:**
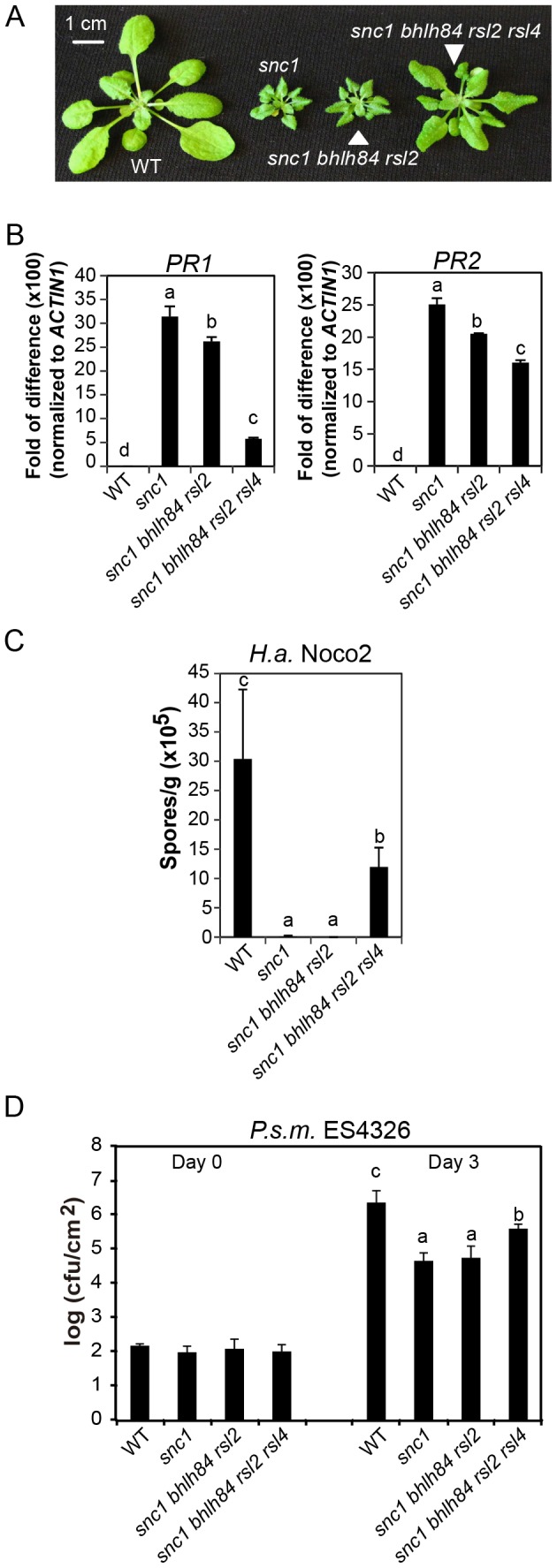
*bhlh84 rsl2 rsl4* partially suppresses the autoimmunity of *snc1*. A. Morphology of WT, *snc1*, *snc1 bhlh84 rsl2*, and *snc1 bhlh84 rsl2 rsl4* quadruple mutant. B. Relative *PR1* and *PR2* gene expression in the indicated genotypes was determined by real-time PCR. The experiment was carried out as in Figure 1C except that the plants used were two-week-old. Statistical differences among the indicated genotypes were analyzed by a StatsDirect software, which are indicated using different letters (*p*<0.05). C. Growth of *H.a.* Noco2 on the indicated genotypes was measured and analyzed using a similar method as used in Figure 1D, except that spores were collected at 8 days post inoculation. D. Bacterial growth of *P.s m.* ES4326 on four-week-old plants of the indicated genotypes at 0 and 3 days post-inoculation with bacterial inoculum of OD_600_ = 0.001. Bars represent means of five replicates ±SD. Statistically different groups were analyzed by StatsDirect and labelled by different letters (p<0.005).

To investigate the biological function of *bHLH84* and its paralogs in *snc1*-mediated immunity, we crossed *bhlh84 rsl2* with *snc1* and isolated triple mutant *snc1 bhlh84 rsl2*. The dwarf phenotype of *snc1* was not suppressed in the triple mutant (Figure 3A). We further crossed *snc1 bhlh84 rsl2* with *rsl4 rsl2* [29] and isolated quadruple mutant *snc1 bhlh84 rsl2 rsl4* from the F2 generation by genotyping *bhlh84*, *rsl4* and *snc1* loci. The quadruple mutant plants were significantly larger than those of *snc1* (Figure 3A). Consistent with the morphological suppression, the expression of *PR1* and *PR2* in the quadruple mutant was significantly decreased compared to *snc1* plants while only slight reduction was observed in the triple mutant (Figure 3B). In addition, when the quadruple mutant seedlings were challenged with *H.a.* Noco2 and *P.s.m.* ES4326, more pathogen growth was observed compared to *snc1*, although the resistance was not restored to wild type levels (Figure 3C and 3D). Taken together, the *bhlh84 rsl2 rsl4* triple mutant partially suppresses *snc1*, suggesting that *bHLH84* and its paralogs are functionally redundant and required for the autoimmunity of *snc1*.

When we further isolated *snc1 rsl2 rsl4* (Figure S3A and S3B), the triple mutant was slightly larger than *snc1*. Since *snc1 bhlh84 rsl2* plants were indistinguishable from *snc1* in size, it can thus be concluded that these three TFs are not equally redundant; *RSL4* seems to play a slightly larger role than bHLH84 in *snc1*-mediated autoimmunity.

### Overexpression of *bHLH84*, *RSL2*, or *RSL4* exhibits extreme dwarfism likely due to autoimmunity

**Figure 4:**
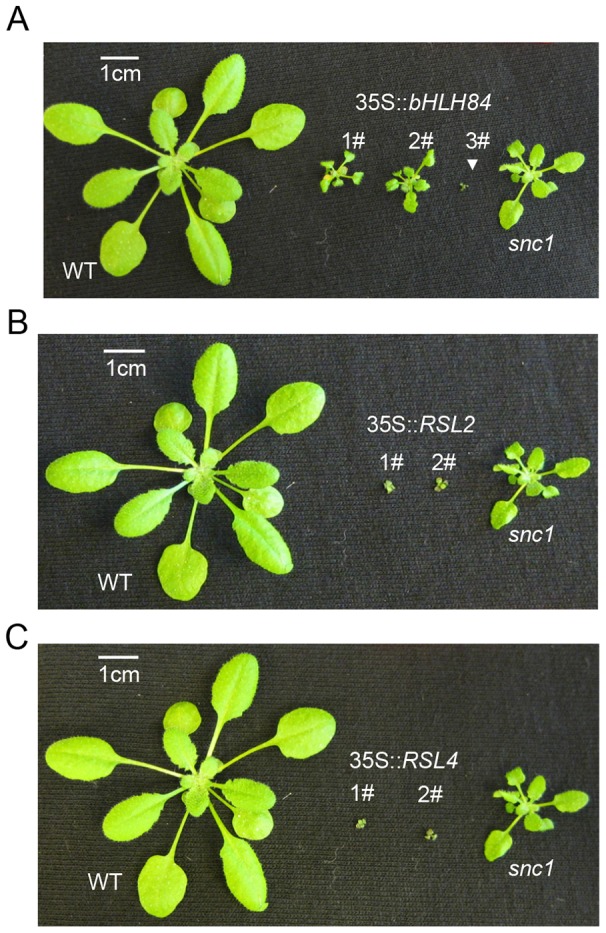
*bHLH84*, *RSL2* and *RSL4* all exhibit dwarfism when overexpressed. A. Morphology of WT, three representative T1 transgenic plants of *35S:bHLH84* in Col-0, and *snc1.* B. Morphology of WT, two representative T1 transgenic plants of *35S:RSL2* in Col-0, and *snc1*. C. Morphology of WT, two representative T1 transgenic plants of *35S:RSL4* in Col-0, and *snc1*. All pictures were taken from four-week-old soil-grown plants.

To further test the redundant roles of bHLH84 and its paralogs, we overexpressed *bHLH84*, *RSL4* or *RSL2* in Col-0 by transforming plants with the coding sequence of each gene without any epitope tags under the control of the *35S* promoter. When screening T1 populations, multiple plants with extremely dwarf morphology were observed for each genotype (Figure 4). Intriguingly, plants of intermediate sizes were observed in the transgenic lines overexpressing *bHLH84*, while the majority of the plants overexpressing *RSL4* or *RSL2* were tiny and gradually perished, presumably as a result of extreme autoimmunity. The phenotypic similarity in these overexpression progeny further supports the functional redundancy among these three TFs in regulating plant immunity.

### SNC1 contributes to the constitutive activation of defense responses in *OXbHLH84-GFP-HA* transgenic plants

**Figure 5:**
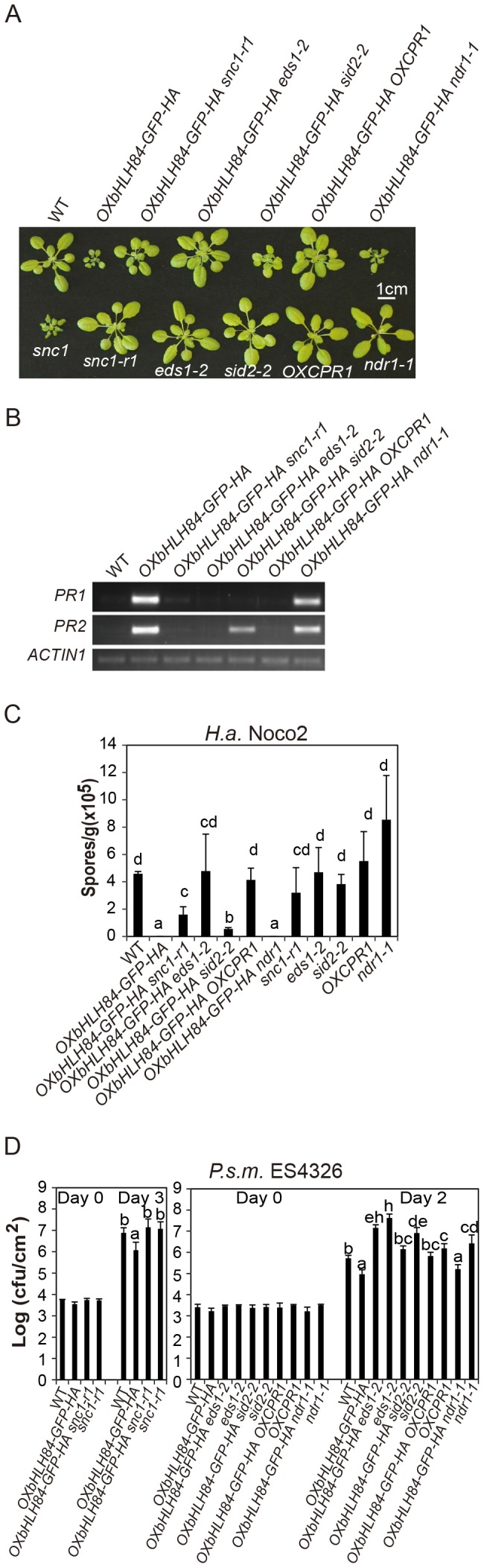
Epistasis analysis between *OXbHLH84-GFP-HA* and *snc1-r1*, *eds1-2*, *sid2-2*, *OXCPR1*, and *ndr1-1*. A. Morphology of four-week-old soil-grown plants of the indicated genotypes. B. *PR1* and *PR2* gene expression of the indicated genotypes as determined by RT-PCR. The experiment was carried out as in Figure 1C except that gene expression was determined by RT- PCR. C. Quantification of *H.a*. Noco2 sporulation on the indicated genotypes using the same method as in Figure 1D (*p*<0.05). D. Bacterial growth of *P.s.m.* ES4326 on four-week-old plants of the indicated genotypes at 0 and 2/3 days post-inoculation with bacterial inoculum of OD_600_ = 0.001. Bars represent means of five replicates ±SD. The same statistical analysis method as described in Figure 3D was used (*p*<0.05). The experiment was repeated three times with similar results.

As with *snc1*, the dwarf morphology of *OXbHLH84-GFP-HA* plants was largely suppressed when grown at 28°C (Figure S4) [30]. This observation led us to ask whether SNC1 is required for the autoimmunity of *OXbHLH84-GFP-HA*. As shown in Figure 5A, the *snc1-r1* allele (a loss-of-function allele of *SNC1* in which 8 bp of the first exon of *SNC1* is deleted from fast neutron mutagenesis; [20]) could largely suppress the dwarf morphology of *OXbHLH84-GFP-HA*. Consistent with the observed morphological suppression, defense response phenotypes conferred by *OXbHLH84-GFP-HA*, including up-regulation of *PR* gene expression and resistance to *P.s.m.* ES4326 and *H.a.* Noco2, were significantly suppressed by *snc1-r1* (Figures 5B, 5C and 5D), indicating that a functional SNC1 is indispensable for the effects of *bHLH84* overexpression. As CPR1 (CONSTITUTIVE EXRPRESSER OF *PR* GENES 1) targets SNC1 for degradation [31], we crossed *OXbHLH84-GFP-HA* with plants overexpressing *CPR1* (*OXCPR1*). The dwarf morphology and enhanced resistance of *OXbHLH84-GFP-HA* were largely suppressed (Figure 5), providing further support that SNC1 contributes to the autoimmune phenotypes associated with *OXbHLH84-GFP-HA*. In addition, the bHLH84-GFP-HA protein level in *snc1-r1* or *OXCPR1* background was not changed (Figure S5), suggesting that SNC1 does not affect bHLH84 protein accumulation.

### Epistasis analysis reveals that constitutive activation of defense responses in *OXbHLH84-GFP-HA* is *EDS1-* and *SID2-* dependent and *NDR1-* independent

To further dissect the function of *bHLH84* in plant defense pathways, *OXbHLH84-GFP-HA* was crossed with various mutants of key components in plant immunity, including *eds1-2*, *sid2-2*, and *ndr1-1* [8], [32], [33]. As shown in Figure 5A, *eds1-2* and *sid2-2* could fully and partially suppress the morphology of *OXbHLH84-GFP-HA* in terms of leaf shape and plant size, respectively, while *ndr1-1* had little effect. The enhanced *PR* gene expression and resistance to *H.a.* Noco2 and *P.s.m.* ES4326 were fully suppressed by *eds1-2* and partially by *sid2-2* (Figure 5B, 5C and 5D), indicating that *EDS1* and SA are required for the autoimmunity in *OXbHLH84-GFP-HA*. In contrast, *ndr1-1* was not able to suppress the enhanced *PR* gene expression, *H.a*. Noco2 and *P.s.m.* ES4326 resistance conferred by *OXbHLH84-GFP-HA*, indicating that the constitutive activation of defense responses in *OXbHLH84-GFP-HA* is *NDR1*-independent.

### *bHLH84* does not directly regulate *SNC1* transcription

**Figure 6:**
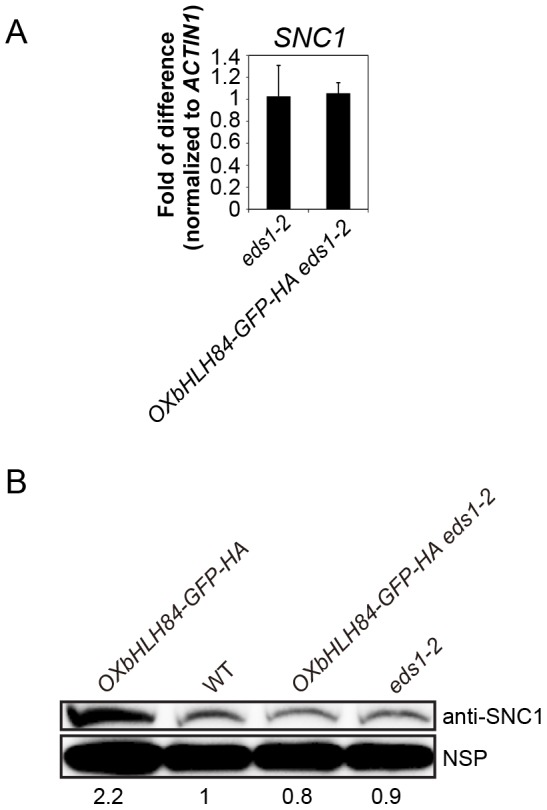
bHLH84 does not regulate *SNC1* transcription. A. *SNC1* expression level in *eds1-2* and *OXbHLH84-GFP-HA eds1-2* plants as determined by real-time PCR. *SNC1* transcripts were amplified from cDNA, with primers specific to *SNC1* cDNA by real-time PCR. The value of *SNC1* expression for each genotype was normalized to that of *ACTIN1*. The value of each genotype was normalized to that of *eds1-2*. Bars represent means of three replicates ±SD. Statistical differences among the indicated genotypes were analyzed by StatsDirect software, which are represented using different letters (*p*<0.05). B. SNC1 protein levels in *OXbHLH84-GFP-HA*, WT, *OXbHLH84-GFP-HA eds1-2* and *eds1-2* plants. Total protein was extracted from leaves of four-week-old soil-grown plants. SNC1 protein levels were examined by immunoblot using an anti-SNC1 antibody [62]. NSP, a non-specific protein band that was used as internal loading control. Image J was used to quantify the band intensities of SNC1 and NSP. The band intensity of SNC1 relative to NSP was calculated for each genotype and normalized to the value of WT. The amount of SNC1 relative to WT is shown at the bottom of each genotype.

As *SNC1* is required for the constitutive activation of the defense responses of *OXbHLH84-GFP-HA* plants, we asked whether *bHLH84* could directly regulate *SNC1* transcription. We observed that the transcription and protein levels of SNC1 in *OXbHLH84-GFP-HA* plants were slightly higher than in WT (Figure S6). However, this up-regulation of *SNC1* is probably due to the positive feed-back effect resulting from the high SA in the autoimmune transgenic plants [34]. To avoid interference from the feed-back up-regulation of *SNC1*, we used *OXbHLH84-GFP-HA eds1-2* plants to examine *SNC1* transcription level. Real-time PCR showed that no significant change in *SNC1* transcription was detected in *OXbHLH84-GFP-HA eds1-2* compared to *eds1-2* control plants (Figure 6A). As a consequence, the SNC1 protein level in *OXbHLH84-GFP-HA eds1-2* was similar to that of *eds1-2* (Figure 6B). In addition, we tested the transcript levels of selected *R* genes including *RPS6*, *RPS4*, *RPP2*, *RPP4*, *RPS2*, *RPS5*, and *RPM1* in the *OXbHLH84-GFP-HA eds1-2* background. Similar to *SNC1*, none of the tested *R* genes showed over 1.2-fold transcriptional changes when compared to *eds1-2* (Figure S7A). In addition, no significant up-regulation of *R* genes was observed in *OXbHLH84-GFP-HA snc1-r1* double mutant compared to *snc1-r1* control plants (Figure S7B). Taken together, *bHLH84* does not seem to participate in the direct transcriptional regulation of *SNC1* or other tested *R* genes, unless bHLH84 recruits both EDS1 and SNC1 for this regulation.

### bHLH84 interacts with SNC1 and RPS4 *in planta*

**Figure 7:**
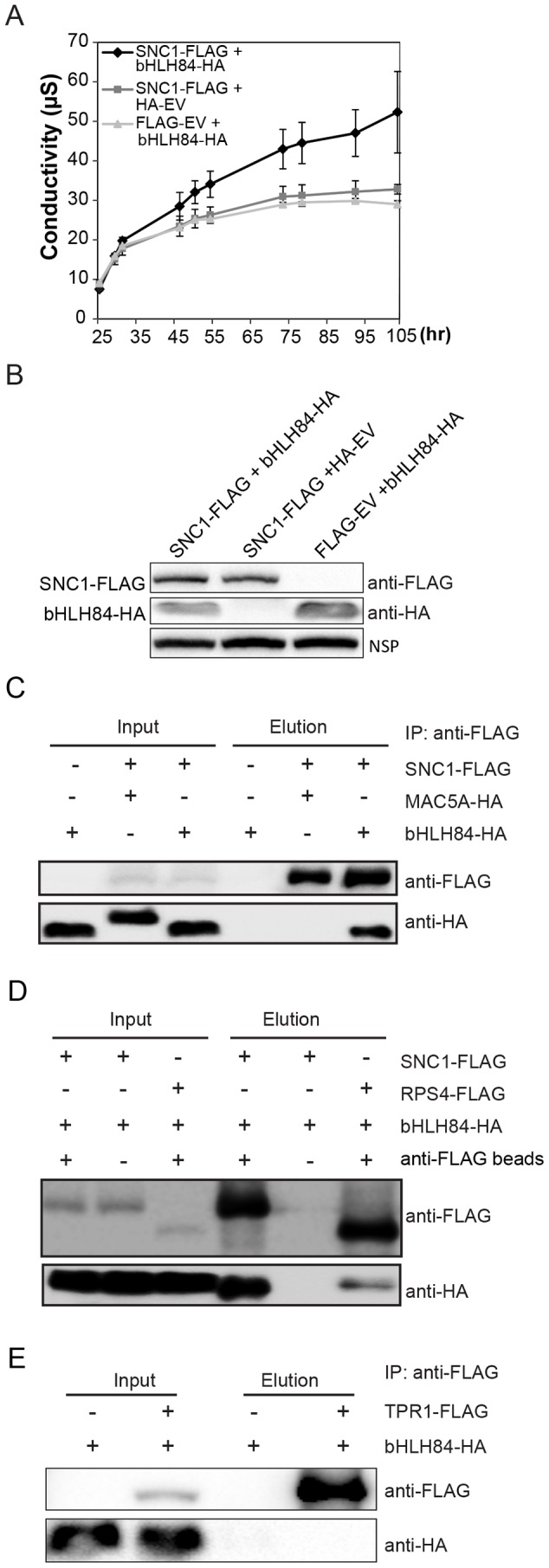
bHLH84 interacts with SNC1 or RPS4 *in planta*. A. Ion leakage as measured in *N. benthamiana* leaves infiltrated by *Agrobacterium* containing the indicated constructs. *Agrobacterium* containing *pCambia1300-35S* -FLAG or *pCambia1300-35S-* HA empty vectors (EV) served as control. Leaf disc samples were collected at different time points post infiltration. Bars represent means of three replicates ±SD. For each replicate, 12 leaf discs were used. B. Protein expression of bHLH84-HA and SNC1-FLAG in *N.benthamiana* leaves with the indicated infiltration. NSP, signals from a non-specific protein band that served as loading control. C. bHLH84-HA co-immunoprecipitates with SNC1-FLAG when co-expressed in *N. benthamiana* leaves. Four-week-old *N. benthamiana* leaves were co-infiltrated with *Agrobacterium* containing *pCambia1300-35S-bHLH84-HA* and *pCambia1300-35S-SNC1-FLAG* at OD_600_ = 0.2 for each strain. *N. benthamiana* leaves co-infiltrated with *Agrobacterium* containing *pCambia1300-35S-FLAG* EV and *pCambia1300-35S-bHLH84-HA* or *pCambia1300-35S-SNC1-FLAG* and *pGreen229-MAC5A-HA* [36] were used as negative controls. 1.5 g of *N. benthamiana* leaf tissues for each infiltration were collected 36 hours post-inoculation and the protein extracts from the leaves were subjected to IP using anti-FLAG beads. Input indicates protein sample before IP. Elution indicates protein sample competitively eluted from the beads by 3×FLAG peptides. D. bHLH84-HA co-immunoprecipitates with RPS4-FLAG when co-expressed in *N. benthamiana* leaves. A similar experimental procedure was carried out as in Figure 7C. Protein sample of SNC1-FLAG and bHLH84-HA incubated with anti-FLAG beads served as positive control while proteins incubated with protein A agarose beads without the conjugated anti-FLAG antibody served as negative control. E. bHLH84-HA did not co-immunoprecipitate with TPR1-FLAG when co-expressed in *N. benthamiana* leaves. A similar experimental procedure was carried out as in Figure 7C except that *pCambia1305-ProTPR1-TPR1-FLAG* was used instead of *pCambia1300-35S-SNC1-FLAG*.

As the dependence of *OXbHLH84-GFP-HA* on a functional SNC1 and the partial suppression of *snc1* by *bhlh84 rsl2 rsl4* resembles the genetic interactions between SNC1 and TPR1/MOS10, and SNC1 interacts with TPR1 [17], we further tested whether bHLH84 associates with SNC1. We attempted a nuclear co-immunoprecipitation (co-IP) experiment using *OXbHLH84-GFP-HA* transgenic plants, which carry C-terminal GFP and HA double tags. Unfortunately, we were unable to detect the bait after immunoprecipitation in the elution, while all the proteins were found in the flow-through fraction (Figure S8A). As an alternate approach, we transformed Arabidopsis plants with a construct expressing *bHLH84* under its native promoter and containing an N-terminal GFP tag. The protein produced was functional, as the transgenic plants resembled the original *OXbHLH84-GFP-HA* plants (Figure S8B). However, when they were used for co-IP with anti-GFP beads, the bait still could not be pulled down (Figure S8C). The inability of bHLH84 to be pulled down using immunoprecipitation could be due to unknown structural complexity of the protein. Since we were not able to carry out a co-IP experiment with bHLH84 as bait using epitope-tagged *bHLH84* transgenic plants, we decided to examine the interaction between SNC1 and bHLH84 using the *Nicotiana benthamiana* transient expression system [35]. Interestingly, when both proteins were expressed in *N. benthamiana* leaves, we consistently observed a faster hypersensitive response (HR), which was obvious a few hours earlier compared to when SNC1-FLAG was expressed with the control vector (Figure S9A and S9B). This was further confirmed by the ion leakage analysis of the infiltrated leaves (Figure 7A). Both proteins were expressed efficiently in *N. benthamiana* (Figure 7B). When co-immunoprecipitation was carried out, SNC1-FLAG could specifically pull down bHLH84-HA, but not an unrelated nuclear protein MAC5A-HA (Figure 7C, [36]), indicating that bHLH84 can interact with SNC1 *in planta*.

As bHLH84 is able to interact with SNC1 *in planta*, we further examined the interaction specificity between bHLH84-HA and other R proteins by conducting co-IP of bHLH84-HA with either RPS4-FLAG, RPS2-FLAG or RPS6-FLAG. As shown in Figure 7D, RPS4-FLAG could also immunoprecipitate bHLH84-HA, although not as efficiently as SNC1-FLAG. However, RPS2-FLAG or RPS6-FLAG could not pull down bHLH84-HA (Figure S10). Taken together, bHLH84-HA can specifically interact with SNC1-FLAG or RPS4-FLAG *in planta*.

SNC1 was previously shown to interact with transcriptional co-repressor TPR1, which does not contain a DNA binding domain [17]. Additionally, the SNC1-dependent phenotypes observed upon overexpressing bHLH84 are similar to those observed when TPR1 is overexpressed. We therefore asked whether bHLH84 interacts with TPR1. As shown in Figure 7E, bHLH84-HA could not be pulled down by TPR1-FLAG, indicating that bHLH84 does not interact with TPR1 *in planta*. In addition, when we co-expressed SNC1-FLAG, bHLH84-HA and TPR1-HA in *N. benthamiana*, SNC1-FLAG was able to pull down both TPR1-HA and bHLH84-HA (Figure S11). The IP efficiency of TPR1-HA by SNC1-FLAG with all three proteins expressed was comparable to that with only TPR1-HA and SNC1-FLAG expressed. On the other hand, the IP efficiency of bHLH84-HA by SNC1-FLAG varied from trial to trial. Taken together, these data suggest that the interactions of SNC1-bHLH84 and SNC1-TPR1 *in planta* are independent, although whether there is competition between bHLH84 and TPR1 in associating with SNC1 is unclear.

To further investigate whether bHLH84 is able to directly interact with SNC1, we carried out yeast-two-hybrid experiment by co-transforming bHLH84 fused with AD and SNC1 fused with BD. Since we failed in making a full-length SNC1 construct, we made truncated SNC1 segments. As shown in Figure S12, yeast cells transformed with bHLH84-AD and different truncated SNC1 fused with BD were not able to grow on the selection plates, suggesting that bHLH84 does not directly interact with the truncated SNC1 segments in yeast. Moreover, the interaction between bHLH84 and SNC1 probably demands a properly folded full-length SNC1 or an intermediate partner. As EDS1 is required for the function of bHLH84 and EDS1 was shown to interact with SNC1 [37], we asked whether EDS1 or its interacting protein PAD4 [7] might be the intermediate partner. However, we did not detect interaction between bHLH84 and EDS1or bHLH84 and PAD4 (Figure S13), suggesting that EDS1or PAD4 is not likely mediating the interaction between SNC1 and bHLH84.

## Discussion

From a targeted reverse genetic screen, we have identified a group of TFs, bHLH84 and its paralogs RSL2 and RSL4, which serve as transcriptional regulators for plant immunity. bHLH84 constitutively activates defense responses when overexpressed, and this activation is *SNC1*-dependent. bHLH84 was further demonstrated to be a transcriptional activator. In addition, the autoimmune phenotypes of *snc1* can be partially suppressed by *bhlh84 rsl2 rsl4* triple mutant, suggesting that *bHLH84* and *SNC1* are mutually dependent. bHLH84 does not seem to directly regulate the transcription of *SNC1* or other tested *R* genes. However, the specific interaction between bHLH84 and NLRs including SNC1 and RPS4 *in planta* suggests that it associates with nuclear NLRs to mediate downstream transcriptional reprogramming. As we failed to observe association between bHLH84 and the repressor protein TPR1 which also interacts with SNC1, we propose that bHLH84 activates defense responses by forming a complex with SNC1 that functions in parallel with the SNC1-TPR1 complex to activate downstream positive regulators (Figure S14).

### The targeted reverse genetic screen is a useful approach to identify new players in biological pathways

Previous work on MLA, N, RRS1 and SNC1 suggests that the interactions between some nuclear R proteins and their associating TFs are essential in regulating defense responses [9], [11], [12], [15], [17], [18]. Different approaches have been utilized to isolate TFs that are able to interact with nuclear R proteins. TPR1, which associates with SNC1 to repress negative regulators of immunity, was isolated from a forward genetic screen for suppressors of *snc1* [17]. Yeast-two-hybrid screens have been successfully used to identify TFs in plant immunity. For example, SPL6 was initially identified from a yeast-two-hybrid screen and was further confirmed to interact with N in tobacco [18]. In addition, identified from yeast-two-hybrid screens, MYB6 and WRKY1 were shown to interact with MLA in barley to initiate disease resistance signaling in an antagonistic manner [15]. In this study, we used an alternative reverse genetic screen and successfully identified a group of novel TFs that play critical roles in plant immunity.

Our targeted reverse genetic approach has several advantages. Since plant defense to UV radiation is regulated by many of the same factors as pathogen resistance [23]–[25], while UV treatment datasets exclude a large number of genes that are manipulated by pathogen effectors which are not directly related to defense responses [38], the number of target genes we chose from the UV-induced database is more manageable for a reverse genetics screen. All the selected TFs were overexpressed in both Col-0 and *snc1* backgrounds, facilitating rapid identification of both defense enhancers and suppressors (Table S1). Furthermore, the functional redundancy predicament often encountered in forward genetic screens can be effectively avoided by using the overexpression approach. Finally, our approach evades self-activation problems that are often associated with yeast-two-hybrid screens for transcriptional activators. Specifically, bHLH84 exhibits strong self-activation when fused with GAL4 binding domain in yeast (data not shown), thus cannot be identified from a yeast-two-hybrid screen. However, our screen does rely on the availability of high-quality microarray data, which may still overlook TFs with relatively low expression level changes.

### bHLH84 functions as a transcriptional activator that is able to bind N1- or N2-boxes

As bHLH84 was shown to be a transcriptional activator, we attempted chromatin immunoprecipitation (ChIP) to identify target genes of bHLH84. However, as with our co-IP experiments (Figure S8), the bHLH84-GFP-HA protein could not be pulled down when subjected to ChIP (Figure S15). Thus we were unable to identify the target DNA of bHLH84 *in planta*. Using yeast-one-hybrid assay as an alternative approach, we attempted to identify the DNA-binding sequences of bHLH84. Many bHLH type TFs were shown to bind sequences containing a consensus core element E-box (5′-CANNTG-3′), with the palindromic G-box (5′-CACGTG-3′) being the most typical form [39]. Some bHLH proteins bind to non-E-box sequences (N-box), such as 5′-CACGc/aG-3′ and 5′-CGCGTG-3′ [40], [41]. As shown in Figure S16A and Figure S16B, compared with the bHLH84 alone or cis-element alone negative controls, the most enhanced yeast growth was observed on SD-Leu-Trp-His media when AD-bHLH84 was co-transformed with pHIS2-N1-box, while considerably enhanced growth was observed when AD-bHLH84 was co-transformed with pHIS2-N2-box. No enhanced yeast growth was observed in G-box or N3 box co-transformations. These data suggest that bHLH84 is able to bind N1- and N2-boxes, but not N3- or G-boxes. These data are consistent with the prediction that TFs in this bHLH subfamily are non E-box binders [42]. Although the potential binding sites of bHLH84 have been revealed, it is still difficult to predict its target genes. More sophisticated ChIP experiments designed in the future may be able to solve this problem.

### bHLH84 and its paralogs are implicated in plant immunity

The bHLH-containing proteins constitute a large conserved TF family in eukaryotes [43], [44]. They have been studied intensively in yeast and humans, providing evidence for their regulatory functions in cell proliferation and cellular differentiation pathways [45]–[48].

While only a few bHLH proteins have been studied in detail in plants, they have been shown to serve regulatory functions in multiple biological pathways. For example, a group of bHLH TFs in *Zea mays* regulate the production of the purple anthocyanin pigments by interacting with R2R3-MYB TFs [49]. In Arabidopsis, GL3 (GLABRA3) regulates trichome development through its interaction with MYB-like TF GL1(GLABRA1) [50]. Another small subfamily of bHLH TFs, referred to as phytochrome-interacting factors (PIFs), have been shown to play diverse functions including regulating light signaling pathways, seed germination, seedling photomorphogenesis, and shade avoidance responses via their interactions with phytochromes [51]–[56]. In addition, JAM1 (ABA-INDUCIBLE BHLH-TYPE TRANSCRIPTION FACTOR/JA-ASSOCIATED MYC2-LIKE), acts as a transcriptional repressor and negatively regulates JA signaling [57]. bHLH84 and its paralogs have previously been shown to regulate root hair elongation [29], [58]. However, they are the first few bHLH TFs found to be involved in plant immunity. Since bHLH TFs form one of the largest TF families in plants, it is difficult to imagine that these three TFs are the only bHLHs involved in immune regulation. Lethality of the knockout mutants or redundancy could be the factors prohibiting others from being discovered. Future novel methods, such as our overexpression approach, may facilitate the functional studies of more TFs in large families.

### bHLH84 and its paralogs function redundantly in NLR-mediated immunity

As one of the largest TF families in Arabidopsis with 147 members, bHLH TFs are further subdivided into 12 major subfamilies based on sequence similarity. bHLH84 and its paralogs belong to the VIIIc subgroup [28]. In this study, we have experimentally shown that bHLH84, RSL2 and RSL4 redundantly regulate defense responses. Overexpression of any of these proteins results in constitutive activation of defense responses (Figure 4). Their redundancy was further demonstrated using the triple mutant of *bhlh84 rsl4 rsl2*, which is able to partially suppress the autoimmune phenotypes of *snc1* (Figure 3), and compromise RPS4-mediated defense responses (Figure 2E). It is possible that additional members of the VIIIc subfamily are also functionally redundant with bHLH84. Future construction of higher order *bhlh* mutants may provide insight into the additional redundant relationships among these family members.

Typically, the bHLH domain contains approximately 60 amino acids and is comprised of a stretch of hydrophilic and basic residues at the N terminus, followed by two amphipathic alpha-helices connected by an intervening loop [44]. The helix-loop-helix and the basic region of the bHLH are required for DNA-binding, whereas the helix-loop-helix region alone often enables homo- or heterodimerization with other bHLH proteins. Since the single mutants of *bhlh84*, *rsl2* and *rsl4* do not exhibit obvious phenotypes, we speculate that if dimerization occurs, it would most likely be homodimerization rather than heterodimerization. The dimerized bHLH84 or its paralogs may bind to the same DNA region, thus regulating immunity in a similar manner. In addition, bHLH TFs often associate with other types of TFs, including MYBs and bZIPs for transcriptional reprogramming [49], [56], thus we cannot exclude the possibility that there are more unknown TFs that are also involved in the bHLH84-SNC1 complex.

As the expression level of *SNC1* is comparable in *eds1-2* and *OXbHLH84-GFP-HA eds1-2* backgrounds (Figure 6), bHLH84 does not seem to regulate *SNC1* expression. In addition, we did not observe transcriptional up-regulation of tested *R* genes in *OXbHLH84-GFP-HA snc1-r1* or *OXbHLH84-GFP-HA eds1-2* plants (Figure S7), suggesting that *bHLH84* does not directly regulate the transcription of *R* genes.

As we also detected attenuated immunity against *P.s.t. avrRps4* in *bhlh84 rsl2 rsl4* triple mutant (Figure 2E), and interaction between RPS4 and bHLH84 in *N.benthamina* (Figure 7D), bHLH84 and its paralogs seem to be not just specific to SNC1. As both RPS4's and SNC1's nuclear localizations are critical to their defense activation [10], [14], we speculate that these bHLH TFs may work together with selective nuclear TIR-NB-LRRs to trigger downstream immunity. More in-depth investigations on the interactions of other nuclear TIR-NB-LRR proteins with these TFs might reveal more R proteins working together with these bHLH proteins.

### bHLH84 and TPR1 function in parallel to regulate SNC1-mediated resistance

Overexpression of either *bHLH84* or *TPR1* results in SNC1-dependent autoimmunity, indicating that both bHLH84 and TPR1 positively regulate SNC1-mediated defense responses. Both bHLH84 and TPR1 were shown to associate with SNC1, although no interaction was detected between bHLH84 and TPR1, suggesting that bHLH84-SNC1 and TPR1-SNC1 probably function in distinct complexes (Figure 7, S11 and S14). Their downstream target genes are probably different, as bHLH84 is a transcriptional activator while TPRs are repressors. Defense activation induced by SNC1 is likely achieved through a combination of activation of positive regulators and repression of negative regulators.

## Materials and Methods

### Construction of plasmids

The genomic sequences of selected *TFs*, excluding the stop codon and including approximately 1.5 kb sequence upstream of the start codon, were amplified by PCR with two different restriction enzyme sites separately introduced at the two primer ends. The chosen restriction enzyme sites were KpnI, SalI, SacI, XbaI or PstI. The amplified fragments were then digested and ligated to modified *pCambia1305* vectors harboring C-terminal GFP and HA tags. These constructs were transformed into *snc1* and Col-0 using the floral dip method [26].

For overexpression of *bHLH84*, *RSL2* and *RSL4*, coding sequences of the genes were amplified by PCR with two different restriction enzyme sites separately introduced at the two primer ends. The primer sequences can be found in Table S2. The fragments were then digested and ligated to the *pG229HAN* vector with a *35S* promoter.

For the *pCambia1300-35S-SNC1-FLAG*, *pCambia1300-35S-RPS4-FLAG* and *pCambia1300-35S-RPS6-FLAG* constructs used in the transient expression in *N. benthamiana*, the genomic region of *SNC1*, *RPS4* or *RPS6* without the stop codon, was cloned into the *pCambia1300* vector with a *35S* promoter and a C-terminus FLAG tag. For other *pCambia1300* constructs used in the transient expression, the CDS regions of the genes were cloned into the corresponding vectors. The primer sequences can be found in Table S2

### Transgenic screening

Approximately 0.4 g of T1 transgenic seeds for each construct were first plated on solid MS medium containing 30 µg/ml Hygromycin B. 48 one-week-old transformant seedlings per genotype were selected and subsequently transplanted on soil. Col-0 and *snc1* seeds were planted on solid MS medium without any selection and transplanted on soil at the same time to serve as controls. Among the transgenic plants of each genotype, the transformants which showed varied sizes were kept, and T2 seeds from these plants were planted on Hygromycin B plates to analyze transgene copy number, check for the presence of the transgene and validate the background using primers specific to the *SNC1* locus [20]. The transgenic plants with heritable phenotypes and with the correct backgrounds were then subjected to *H.a.* Noco2 infection to examine whether their altered morphology is correlated with altered resistance. Resistance was scored based on the degree of deviation from that observed in the control plants. More specifically, transgenic plants in Col-0 background showing similar sporulation as Col-0 were scored as no change (NC). Plants showing less sporulation than Col-0 were scored as showing enhanced resistance phenotype with “+”. Plants exhibiting a little sporulation were scored as having more enhanced resistance phenotype with “++”, while the ones showing no sporulation were scored as the most enhanced resistance phenotype as “+++”. For transgenic plants in the *snc1* background, plants showing more sporulation than *snc1* were scored as suppressing phenotype with “−”, while the ones showing less sporulation than *snc1* were scored as enhancing phenotype with “+”.

### Confocal microscopy

Leaves from one-week-old seedlings were soaked in 1 mg/mL (1∶1 [g/v]) propidium iodide (PI) for 3 minutes and rinsed briefly with water before visualization. Root tissues were submerged in 1 µg/ml (1∶1 [g/v]) PI for 10 seconds and mounted in water. For GFP and PI visualization, a Nikon ECLIPSE 80i Confocal microscope was used under 488 nm and 543 nm filter sets.

### Transient protein expression and co-immunoprecipitation in *N. benthamiana*

Transient protein expression in *N. benthamiana* was carried out as previously described [35]. The IP protocol was modified from [59]. Briefly, *Agrobacteria* containing the binary vector *pCambia1300* constructed with the target genes and tags were cultured in LB media with kanamycin selection at 28°C overnight. The bacteria were inoculated into a new culture media (10.5 g/L K_2_HPO_4_, 4.5 g/L KH_2_PO_4_, 1.0 g/L (NH_4_)_2_SO4, 0.5 g/L NaCitrate, 1 mM MgSO_4_, 0.2% glucose, 0.5% glycerol, 50 µM acetosyringone, and 10 mM N-morpholino-ethanesulfonic acid (MES) (pH 5.6), 50 µg/mL Kanamycin) by 1∶50 dilution and cultured for a further 8–12 hours. The bacteria were then harvested by centrifugation at 4000 rpm for 10 minutes and resuspended in MS buffer (4.4 g/L MS, 10 mM MES, 150 µM acetosyringone) to a final concentration of OD_600_ = 0.2 for infiltration into four-week-old *N. benthamiana* leaves.

For co-immunoprecipitation, 3 g of *N. benthamiana* leaves were collected at 36 hours post-infiltration and ground into fine powder in liquid nitrogen using a cold mortar and pestle. The powder was mixed with 6 ml extraction buffer (10% glycerol, 25 mM Tris pH 7.5, 1 mM EDTA, 150 mM NaCl, 10 mM DTT, 2% w/v PVPP, protease inhibitor cocktail) and homogenized by further grinding. All the following steps were carried out at 4°C. The samples were centrifuged at 15000 g for 10 minutes and the supernatants were transferred to new tubes. These two steps were repeated twice before NP40 (Nonidet P-40 Substitute) was added into each supernatant to a final concentration of 0.15%. 30 µl pre-washed protein A or protein G agarose beads were added into each supernatant and incubated for 30 minutes. The mixtures were centrifuged at 4000 rpm for 2 minutes to remove the beads. Each supernatant was incubated with 30 µl anti-FLAG beads or protein A agarose beads for 3 hours, and the beads were pelleted down by centrifuging at 8000 rpm for 1 minute and washed 8 times using extraction buffer containing 0.15% NP40. Proteins specifically bound to the beads were competitively eluted using 100 µl 250 µg/ml 3×FLAG peptides. All the samples were boiled in SDS loading buffer for 5 minutes before running on SDS-PAGE gel.

### Arabidopsis protoplast transient assay for transcriptional activity

The isolation and transfection of Arabidopsis protoplasts and the reporter gene assay were previously described in [27]. Briefly, the Arabidopsis protoplasts were transfected with the reporter construct, the effector construct and the internal control construct as illustrated in Figure 2A. GUS expression was determined using MUG assay (Acros Organics from Fisher Scientific). Fluorescence was measured using a fluorescence spectrophotometer (360/460 nm). The internal LUC expression was examined using a Dual-Luciferase reporter assay system (Promega, E1910).

### Ion leakage assay

The ion leakage assay was performed as previously described [60], with a few modifications. Briefly, twelve leaf discs (7 mm in diameter) per measurement were punched from the infiltrated area at 23 hr post infiltration and placed in a 60 mm petri dish containing 10 ml of ddH_2_O. After 30 minutes, the water was removed and another 10 ml of ddH_2_O was added into the petri dish containing the leaf discs. Conductivity was measured using a 545 Conductivity Multi-purpose Cell (VWR Scientific) at the indicated time points.

### Yeast-one-hybrid and yeast-two-hybrid assays

For yeast-one-hybrid assay, the *pHIS2* derivatives (harboring the N1-, N2-, N3- and G-box cis-elements) were co-transformed with the construct of *pAD-bHLH84* into the yeast strain Y187. For each co-transformation of *pAD-bHLH84* and *pHIS2* derivatives, yeast cells co-transformed with *pHIS2* empty vector (EV) and *pAD-bHLH84* as well as yeast cells cotransformed with *pAD EV* and the *pHIS2* derivatives were used as negative controls. The positive transformants were isolated from SD-Trp-Leu medium. The transformants were then analyzed on the SD-Trp-Leu-His medium supplemented with 60 mM and 100 mM 3-Amino-1,2,4-Triazole (3AT).

For yeast-two-hybrid assays, the *pGBKT7* derivatives containing various truncated *SNC1* fragments were co-transformed with *pAD-bHLH84* into yeast strain Y1347. *pGBKT7* EV cotransformed with *pAD-bHLH84* was used as a negative control. The positive transformants were isolated from SD-Trp-Leu medium. The transformants were then analyzed on SD-Trp-Leu-His medium supplemented with 3 mM 3AT.

## Supporting Information

Figure S1 **Expression analysis of** ***bhlh84*** **and** ***rsl2*** **knockout mutants.** A. *bHLH84* gene expression in WT and *bhlh84* as detected by *bHLH84*-specific primers using RT-PCR. B. *RSL2* gene expression in WT and *rsl2* as detected by *RSL2*-specific primers. The RNA extraction and cDNA preparation in Figure S1A and S1B were carried out as described in Figure 1C. The *ACTIN1* expression served as loading control. The primer information can be found in Table S2.
(PDF)

Figure S2 ***bHLH84*** **and its two close paralogs are not required for RPS6-mediated disease resistance.** Bacterial growth of the avirulent pathogen *P.s.t. hopA1* on plants of the indicated genotypes. The same experimental procedure and statistical analysis were carried out as in Figure 2E.
(PDF)

Figure S3 **The autoimmunity of** ***snc1*** **can be partially suppressed by** ***bhlh84 rsl2 rsl4*** **and marginally suppressed by** ***rsl2 rsl4*** **, but not by** ***bhlh84 rsl2*** **.** A. Morphology of 4-week-old soil-grown plants of the indicated genotypes. B. Fresh weight of 4-week-old soil-grown plants of the indicated genotypes. Six plants were used for each genotype. Statistical differences were analyzed by one-way analysis using StatsDirect software. Variant letters represent statistical difference among the indicated genotypes. (*p*<0.01).
(PDF)

Figure S4 **The dwarf phenotype of** ***OXbHLH84-GFP-HA*** **plants is temperature-sensitive.** Morphology of plants of the indicated genotypes grown at 28°C (top) or 22°C (bottom). The picture was taken when the plants were 2.5 weeks old.
(PDF)

Figure S5 ***snc1-r1*** **and** ***OXCPR1*** **do not affect bHLH84-GFP-HA protein accumulation.** bHLH84-GFP-HA protein levels in the indicated genotypes as detected by immunoblot using anti-HA antibody. The bands of Rubisco stained by ponceau S served as loading control.
(PDF)

Figure S6 **SNC1 protein accumulates more in** ***OXbHLH84-GFP-HA*** **transgenic plant compared to WT.** A. SNC1 protein level in the indicated genotypes as detected by immunoblot using an anti-SNC1 antibody. NSP indicates the non-specific protein band which served as loading control. B. The transcriptional expression of *SNC1* in WT and *OXbHLH84-GFP-HA* plants. *SNC1* transcripts were amplified from cDNA, with primers specific to *SNC1* region by real-time PCR. The value of *SNC1* expression for each reaction was normalized to *ACTIN1*. The value of each genotype was normalized to that of WT. Bars represent means of three replicates ±SD. * indicates significant differences between the two genotypes as analyzed by unpaired student's t test (*p*<0.05).
(PDF)

Figure S7 **The expression levels of selected** ***R*** **genes in** ***OXbHLH84-GFP-HA eds1-2*** **(A) and** ***OXbHLH84-GFP-HA snc1-r1*** **(B) plants.** The expression levels of the indicated *R* genes were determined by real-time PCR as in Figure 6A using primers specific to the individual *R* genes. The expression of *R* genes in *OXbHLH84-GFP-HA eds1-2* (Figure S7A) was relative to that in *eds1-2*, and the *R* gene expression in *OXbHLH84-GFP-HA snc1-r1* (Figure S7B) was relative to that in *snc1-r1*. The primer sequence information can be found in Table S2.
(PDF)

Figure S8 **Nuclear immunoprecipitation (IP) of bHLH84 with C-terminal HA tag or N-terminal GFP tag in Arabidopsis.** A. Nuclear IP of OXbHLH84-GFP-HA in Arabidopsis. The nuclei of *OXbHLH84-GFP-HA* transgenic plants were isolated and nuclear proteins were extracted and subjected to IP with anti-HA beads, using a previously described method [63]. Input indicates protein sample before IP. Elution indicates eluted protein sample. W8 indicates the 8^th^ wash sample. FT (Flow Through) indicates the unbound proteins. “−” indicates sample without OXbHLH84-GFP-HA, which served as a negative control, while “+” indicates OXbHLH84-GFP-HA sample. IP: immunoprecipitation. IB: immunoblot. B. Morphology of four-week-old soil-grown WT, *snc1*, *OXbHLH84-GFP-HA* and *GFP-bHLH84* epitope-tagged transgenic plants. C. Nuclear IP of bHLH84 with N-terminus GFP tag in Arabidopsis. Similar IP procedure as Figure S8A was carried out except that the negative control was *GFP-bHLH84* sample incubated with anti-HA beads.
(PDF)

Figure S9 **bHLH84-HA accelerates HR in** ***N. benthamiana*** **caused by SNC1-FLAG.** A. Representative leaf HR morphology of *N. benthamiana* at 36 hours and 40 hours post-infiltration of *Agrobacterium*. Four-week-old *N. benthamiana* leaves were co-infiltrated with *Agrobacterium* containing the indicated constructs at OD_600_ = 0.2. Agrobacterium containing *pCambia1300-35S* -FLAG or *pCambia1300-35S* –HA empty vectors (EV) served as controls. The cycles label the infiltrated regions. The red arrow points to the HR symptom caused by the co-infiltration of *Agrobacterium* containing *pCambia1300-35-SNC1-FLAG* and *pCambia1300-35S-* bHLH84-HA. B. The percentage of *N. benthamiana* leaves showing HR after infiltration with *Agrobacterium* containing the indicated constructs at the indicated time point. Six leaves of 3 plants were used for inoculations in a pattern similar to that shown in Figure S9A. At the indicated time point, leaves displaying visible gray and slightly shinny cell death spots were recorded as exhibiting HR symptom. For each treatment, the percentage of HR were calculated using the number of leaves showing HR symptom at the indicated time points divided by the total number of inoculated leaves. More quantitative measurements of ion leakage, which reflects the level of cell death, are presented in Figure 7A.
(PDF)

Figure S10 **bHLH84 does not interact with RPS2-FLAG (A) or RPS6-FLAG (B).** A. bHLH84-HA could not be pulled down by RPS2-FLAG when co-expressed in *N. benthamiana* leaves. Four-week-old *N. benthamiana* plants were co-infiltrated with *Agrobacterium* containing *pCambia1300-35S-bHLH84-HA* and *pCambia 1300-35S-RPS2-FLAG* at OD_600_ = 0.2 for each strain. Total protein extracted from 6 g of *N. benthamiana* leaves collected 33 hours post-inoculation was subjected to IP. Half of the sample was incubated with anti-FLAG beads while the other half was incubated with protein A agarose beads without the conjugated anti-FLAG antibody, which serves as negative IP control. Input indicates protein sample before IP. Elution indicates protein sample competitively eluted from the beads by 3×FLAG peptides. B. bHLH84-HA could not be pulled down by RPS6-FLAG when co-expressed in *N. benthamiana* leaves. Similar procedures were carried out as described in Figure S10A.
(PDF)

Figure S11 **Both bHLH84-HA and TPR1-HA can be immunoprecipitated by SNC1-FLAG when all three protein were co-expressed in** ***N. benthamiana*** **.** Four-week-old *N. benthamiana* leaves were infiltrated with *Agrobacterium* strains carrying the indicated constructs. Total protein extracted from 3 g of *N. benthamiana* leaves for each infiltration collected 36 hours post-inoculation were subjected to IP. Three samples were incubated with anti-FLAG beads while one sample was incubated with protein A agarose beads, which served as negative control. Two western blots of bHLH84-HA from two independent experiments are presented.
(PDF)

Figure S12 **bHLH84 does not interact with truncated SNC1 protein in yeast.** A. Schematic diagram of constructs containing the indicated domains of SNC1.The full-length protein of SNC1 (SNC1-FL) is encoded by 6 exons as shown in the diagram. B. bHLH84 did not interact with the truncated SNC1 segments in yeast-two-hybrid assays. Yeast strain Y1347 cotransformed with *bHLH84* fused with *AD* in *PGADT7* vector and *PGBKT7* constructs with indicated truncated *SNC1* fused with *BD* are plated on SD-Leu-Trp and SD-Leu-Trp-His with 3 mM 3AT. BD-EV indicates the empty PGBKT7 vector. Yeast growth on SD-Leu-Trp indicates successful cotransformation of the constructs and serves as inoculum control.
(PDF)

Figure S13 **bHLH84 does not interact with EDS1 or PAD4 in** ***N. benthamiana*** **.** bHLH84-HA could not be pulled down by EDS1-FLAG or PAD4-FLAG when co-expressed in *N. benthamiana* leaves. Four-week-old *N. benthamiana* plants were co-infiltrated with *Agrobacterium* containing *pCambia1300-bHLH84-HA* and *pCambia 1305-EDS1-FLAG* or *pCambia 1305-PAD4-FLAG* at OD_600_ = 0.2 for each strain. Similar IP procedure was carried out as described in Figure 7D. Protein extracts incubated with protein A agarose beads without anti-FLAG serve as negative control. EDS1-FLAG or PAD4-FLAG are indicated by the arrow. The band in EDS1 elution fraction indicated with an * presumably represents a degradation or cleavage product of EDS1.
(PDF)

Figure S14 **A working model on how SNC1 activates plant immunity through interacting with different transcription factors in distinct protein complexes.** The bHLH84-SNC1 complex may bind to the promoter regions of downstream positive regulators of plant immunity by recognizing the specific cis-elements to activate defense response. This complex most likely contains other unidentified components. It functions separately from and in parallel with the HDA19-TPRs-SNC1 complex, which represses negative regulators of immunity, such as *DND1* and *DND2*, possibly through an unknown DNA-binding protein. “?”s represent unknown protein partners.
(PDF)

Figure S15 **Immunoblot detection of bHLH84-GFP-HA in ChIP (Chromatin Immunoprecipitation) samples.** *bHLH84-GFP-HA* and *snc1* seedlings (10 g of tissue for each genotype) were cross-linked with 1% formaldehyde. Nuclei were extracted, sonicated, and centrifuged to obtain supernatant which was diluted in the ChIP IP buffer and subjected to anti-HA precipitation. Input indicates protein sample before IP. Elution indicates eluted protein sample. “−” indicates sample without OXbHLH84-GFP-HA, which serves as negative control. “+” indicates OXbHLH84-GFP-HA sample. The ChIP experiment was carried out as previously described [17].
(PDF)

Figure S16 **bHLH84 exhibits DNA binding activity with N1- and N2-box cis elements.** A. The sequences of the tested DNA fragments in the yeast-one-hybrid assays. The tripled cis-element sequences are indicated with the underline. B. bHLH84 only exhibited detectable binding activity to N1- and N2-boxes. Yeast cell Y187 cotransformed with constructs harboring *bHLH84* fused with *GAL4* activation domain (AD) and *pHIS2* constructs containing the indicated DNA fragment were plated on the SD-Leu-Trp and SD-Leu-Trp-His with 60 mM or 100 mM 3-Amino-1,2,4-Triazole (3AT). The growth of yeast cells on SD-Leu-Trp-His with 60 mM 3AT or 100 mM 3AT reflects the binding activity of bHLH84 while that on SD-Leu-Trp serves as loading control. Yeast cells cotransformed with *pHIS2* empty vector (EV) and *bHLH84-AD* together with yeast strains cotransformed with *AD* empty vector (EV) and *pHIS2* constructs with the indicated DNA fragments served as negative controls.
(PDF)

Table S1 **Summary of the transcription factors screened and their overexpression phenotypes in** ***snc1*** **and Col-0 background.** In *snc1* background, “−” indicates a suppressing phenotype, and “+” indicates an enhancing phenotype. In Col-0, “+”indicates dwarfism caused by the transgene. “NC” indicates no change. The number of “−” and “+” is correlated with the degrees of the phenotype. Note: A few genes, such as *At2g18670*, *At5g10380* and *At5g67340*, have been recently annotated as RING proteins or U-box proteins. They are more likely to be E3 ligases rather than TFs.
(PDF)

Table S2 **Primers used for genotyping and cloning.**
(XLSX)
