# Supplementary material for: NLR-Associating Transcription Factor bHLH84 and Its Paralogs Function Redundantly in Plant Immunity
Source: PLoS Pathog. 2014 Aug 21;10(8):e1004312. doi: 10.1371/journal.ppat.1004312 (PMC4140859; doi:10.1371/journal.ppat.1004312)
Supplement: Figure S1 — Expression analysis of bhlh84 and rsl2 knockout mutants. A. bHLH84 gene expression in WT and bhlh84 as detected by bHLH84-specific primers using RT-PCR. B. RSL2 gene expression in WT and rsl2 as detected by RSL2-specific primers. The RNA extraction and cDNA preparation in Figure S1A and S1B were carried out as described in Figure 1C. The ACTIN1 expression served as loading control. The primer information can be found in Table S2. (PDF) [file ppat.1004312.s001.pdf]

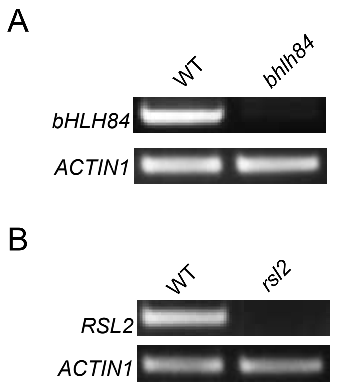

Figure S1. Expression analysis of *bhlh84* and *rsl2* knockout mutants.

A. *bHLH84* gene expression in WT and *bhlh84* as detected by *bHLH84*-specific primers using RT-PCR.

B. *RSL2* gene expression in WT and *rsl2* as detected by *RSL2*-specific primers. The RNA extraction and cDNA preparation in Figure S1A and S1B were carried out as described in Figure 1C. The *ACTIN1* expression served as loading control. The primer information can be found in Table S2.
