# Supplementary material for: NLR-Associating Transcription Factor bHLH84 and Its Paralogs Function Redundantly in Plant Immunity
Source: PLoS Pathog. 2014 Aug 21;10(8):e1004312. doi: 10.1371/journal.ppat.1004312 (PMC4140859; doi:10.1371/journal.ppat.1004312)
Supplement: Figure S2 — bHLH84 and its two close paralogs are not required for RPS6-mediated disease resistance. Bacterial growth of the avirulent pathogen P.s.t. hopA1 on plants of the indicated genotypes. The same experimental procedure and statistical analysis were carried out as in Figure 2E. (PDF) [file ppat.1004312.s002.pdf]

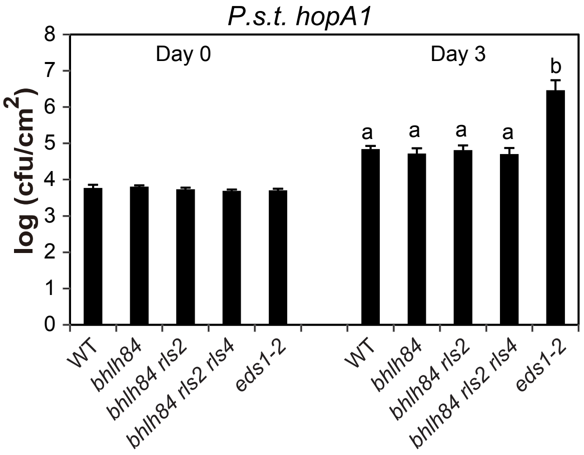


**Figure S2. *bHLH84* and its two close paralogs are not required for RPS6-mediated disease resistance.**

Bacterial growth of the avirulent pathogen *P.s.t. hopA1* on plants of the indicated genotypes. The same experimental procedure and statistical analysis were carried out as in Figure 2E.
