# Supplementary material for: NLR-Associating Transcription Factor bHLH84 and Its Paralogs Function Redundantly in Plant Immunity
Source: PLoS Pathog. 2014 Aug 21;10(8):e1004312. doi: 10.1371/journal.ppat.1004312 (PMC4140859; doi:10.1371/journal.ppat.1004312)
Supplement: Figure S3 — The autoimmunity of snc1 can be partially suppressed by bhlh84 rsl2 rsl4 and marginally suppressed by rsl2 rsl4 , but not by bhlh84 rsl2 . A. Morphology of 4-week-old soil-grown plants of the indicated genotypes. B. Fresh weight of 4-week-old soil-grown plants of the indicated genotypes. Six plants were used for each genotype. Statistical differences were analyzed by one-way analysis using StatsDirect software. Variant letters represent statistical difference among the indicated genotypes. (p<0.01). (PDF) [file ppat.1004312.s003.pdf]

A

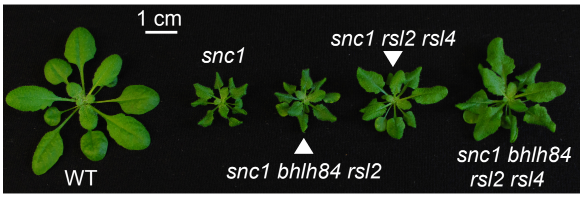

B

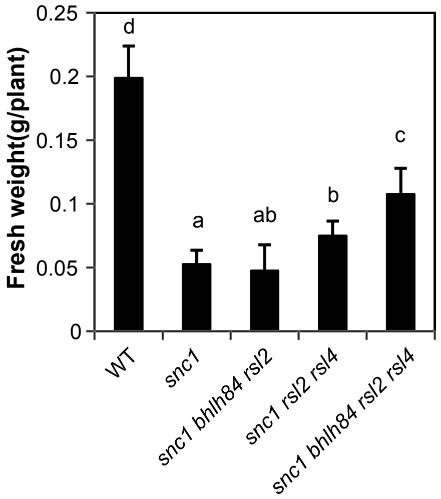

Figure S3. The autoimmunity of *snc1* can be partially suppressed by *bhlh84 rsl2 rsl4* and marginally suppressed by *rsl2 rsl4*, but not by *bhlh84 rsl2*.

A. Morphology of 4-week-old soil-grown plants of the indicated genotypes.
B. Fresh weight of 4-week-old soil-grown plants of the indicated genotypes. Six plants were used for each genotype. Statistical differences were analyzed by one-way analysis using StatsDirect software. Variant letters represent statistical difference among the indicated genotypes. (p<0.01).
