# Supplementary material for: NLR-Associating Transcription Factor bHLH84 and Its Paralogs Function Redundantly in Plant Immunity
Source: PLoS Pathog. 2014 Aug 21;10(8):e1004312. doi: 10.1371/journal.ppat.1004312 (PMC4140859; doi:10.1371/journal.ppat.1004312)
Supplement: Figure S4 — The dwarf phenotype of OXbHLH84-GFP-HA plants is temperature-sensitive. Morphology of plants of the indicated genotypes grown at 28°C (top) or 22°C (bottom). The picture was taken when the plants were 2.5 weeks old. (PDF) [file ppat.1004312.s004.pdf]

Figure S4

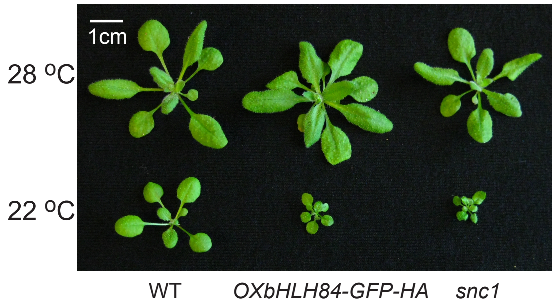

Figure S4. The dwarf phenotype of *OXbHLH84-GFP-HA* plants is temperature-sensitive.

Morphology of plants of the indicated genotypes grown at 28 ºC (top) or 22 ºC (bottom). The picture was taken when the plants were 2.5 weeks old.
