# Supplementary material for: NLR-Associating Transcription Factor bHLH84 and Its Paralogs Function Redundantly in Plant Immunity
Source: PLoS Pathog. 2014 Aug 21;10(8):e1004312. doi: 10.1371/journal.ppat.1004312 (PMC4140859; doi:10.1371/journal.ppat.1004312)
Supplement: Figure S5 — snc1-r1 and OXCPR1 do not affect bHLH84-GFP-HA protein accumulation. bHLH84-GFP-HA protein levels in the indicated genotypes as detected by immunoblot using anti-HA antibody. The bands of Rubisco stained by ponceau S served as loading control. (PDF) [file ppat.1004312.s005.pdf]

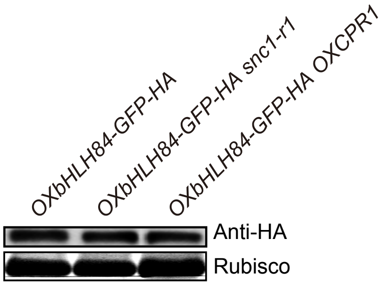


**Figure S5. *snc1-r1* and *OXCPR1* do not affect bHLH84-GFP-HA protein accumulation.**

bHLH84-GFP-HA protein levels in the indicated genotypes as detected by immunoblot using anti-HA antibody. The bands of Rubisco stained by ponceau S served as loading control.
