# Supplementary material for: NLR-Associating Transcription Factor bHLH84 and Its Paralogs Function Redundantly in Plant Immunity
Source: PLoS Pathog. 2014 Aug 21;10(8):e1004312. doi: 10.1371/journal.ppat.1004312 (PMC4140859; doi:10.1371/journal.ppat.1004312)
Supplement: Figure S6 — SNC1 protein accumulates more in OXbHLH84-GFP-HA transgenic plant compared to WT. A. SNC1 protein level in the indicated genotypes as detected by immunoblot using an anti-SNC1 antibody. NSP indicates the non-specific protein band which served as loading control. B. The transcriptional expression of SNC1 in WT and OXbHLH84-GFP-HA plants. SNC1 transcripts were amplified from cDNA, with primers specific to SNC1 region by real-time PCR. The value of SNC1 expression for each reaction was normalized to ACTIN1. The value of each genotype was normalized to that of WT. Bars represent means of three replicates ±SD. * indicates significant differences between the two genotypes as analyzed by unpaired student's t test (p<0.05). (PDF) [file ppat.1004312.s006.pdf]

Figure S6

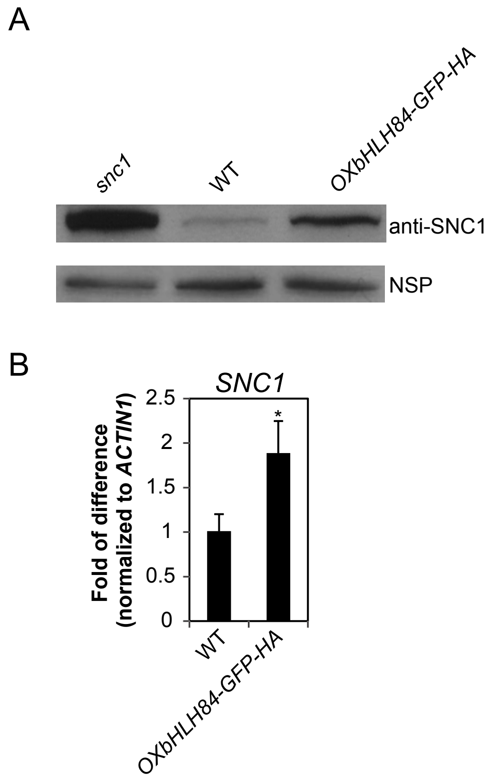

Figure S6. SNC1 protein accumulates more in *OXbHLH84-GFP-HA* transgenic plant compared to WT.

A. SNC1 protein level in the indicated genotypes as detected by immunoblot using an anti-SNC1 antibody. NSP indicates the non-specific protein band which served as loading control.
B. The transcriptional expression of *SNC1* in WT and *OXbHLH84-GFP-HA* plants. *SNC1* transcripts were amplified from cDNA, with primers specific to *SNC1* region by real-time PCR. The value of *SNC1* expression for each reaction was normalized to *ACTIN1*. The value of each genotype was normalized to that of WT. Bars represent means of three replicates ±SD. * indicates significant differences between the two genotypes as analyzed by unpaired student's t test (p<0.05).
