# Supplementary material for: NLR-Associating Transcription Factor bHLH84 and Its Paralogs Function Redundantly in Plant Immunity
Source: PLoS Pathog. 2014 Aug 21;10(8):e1004312. doi: 10.1371/journal.ppat.1004312 (PMC4140859; doi:10.1371/journal.ppat.1004312)
Supplement: Figure S7 — The expression levels of selected R genes in OXbHLH84-GFP-HA eds1-2 (A) and OXbHLH84-GFP-HA snc1-r1 (B) plants. The expression levels of the indicated R genes were determined by real-time PCR as in Figure 6A using primers specific to the individual R genes. The expression of R genes in OXbHLH84-GFP-HA eds1-2 (Figure S7A) was relative to that in eds1-2, and the R gene expression in OXbHLH84-GFP-HA snc1-r1 (Figure S7B) was relative to that in snc1-r1. The primer sequence information can be found in Table S2. (PDF) [file ppat.1004312.s007.pdf]

Figure S7

A

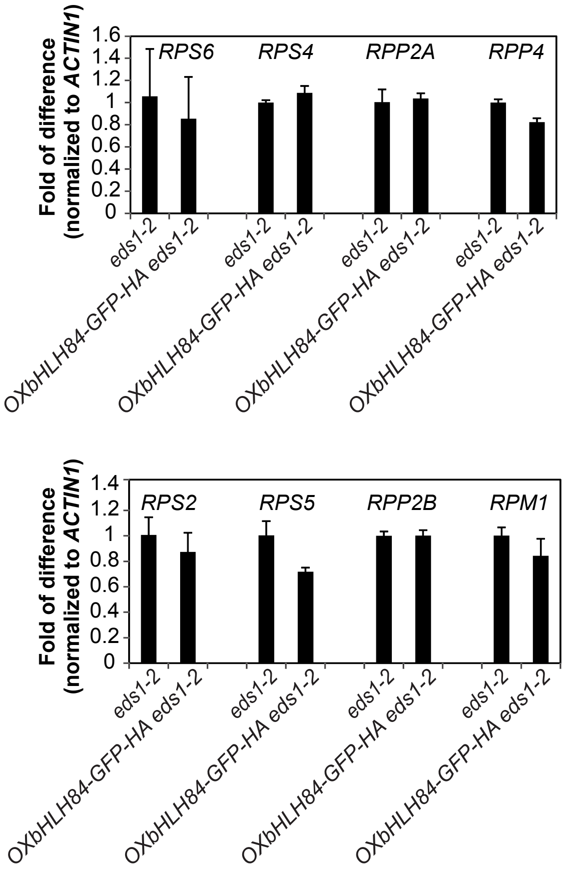
Fold of difference (normalized to ACTIN1)
1.6
1.4
1.2
1
0.8
0.6
0.4
0.2
0
RPS6
RPS4
RPP2A
RPP4
eds1-2
OXbHLH84-GFP-HA eds1-2
Fold of difference (normalized to ACTIN1)
1.4
1.2
1
0.8
0.6
0.4
0.2
0
RPS2
RPS5
RPP2B
RPM1
eds1-2
OXbHLH84-GFP-HA eds1-2

B

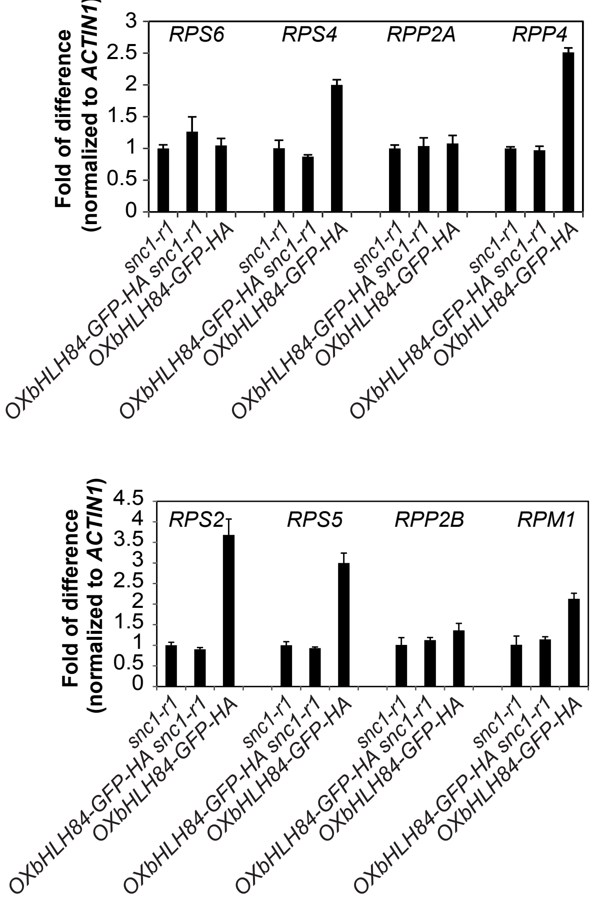
Fold of difference (normalized to ACTIN1)
3
2.5
2
1.5
1
0.5
0
RPS6
RPS4
RPP2A
RPP4
snc1-r1
OXbHLH84-GFP-HA snc1-r1
OXbHLH84-GFP-HA
Fold of difference (normalized to ACTIN1)
4.5
4
3.5
3
2.5
2
1.5
1
0.5
0
RPS2
RPS5
RPP2B
RPM1
snc1-r1
OXbHLH84-GFP-HA snc1-r1
OXbHLH84-GFP-HA

Figure S7. The expression levels of selected *R* genes in *OXbHLH84-GFP-HA eds1-2* (A) and *OXbHLH84-GFP-HA snc1-r1* (B) plants.

The expression levels of the indicated *R* genes were determined by real-time PCR as in Figure 6A using primers specific to the individual *R* genes. The expression of *R* genes in *OXbHLH84-GFP-HA eds1-2* (Figure S7A) was relative to that in *eds1-2*, and the *R* gene expression in *OXbHLH84-GFP-HA snc1-r1* (Figure S7B) was relative to that in *snc1-r1*. The primer sequence information can be found in Table S2.
