# Supplementary material for: NLR-Associating Transcription Factor bHLH84 and Its Paralogs Function Redundantly in Plant Immunity
Source: PLoS Pathog. 2014 Aug 21;10(8):e1004312. doi: 10.1371/journal.ppat.1004312 (PMC4140859; doi:10.1371/journal.ppat.1004312)
Supplement: Figure S8 — Nuclear immunoprecipitation (IP) of bHLH84 with C-terminal HA tag or N-terminal GFP tag in Arabidopsis. A. Nuclear IP of OXbHLH84-GFP-HA in Arabidopsis. The nuclei of OXbHLH84-GFP-HA transgenic plants were isolated and nuclear proteins were extracted and subjected to IP with anti-HA beads, using a previously described method [63]. Input indicates protein sample before IP. Elution indicates eluted protein sample. W8 indicates the 8th wash sample. FT (Flow Through) indicates the unbound proteins. “−” indicates sample without OXbHLH84-GFP-HA, which served as a negative control, while “+” indicates OXbHLH84-GFP-HA sample. IP: immunoprecipitation. IB: immunoblot. B. Morphology of four-week-old soil-grown WT, snc1, OXbHLH84-GFP-HA and GFP-bHLH84 epitope-tagged transgenic plants. C. Nuclear IP of bHLH84 with N-terminus GFP tag in Arabidopsis. Similar IP procedure as Figure S8A was carried out except that the negative control was GFP-bHLH84 sample incubated with anti-HA beads. (PDF) [file ppat.1004312.s008.pdf]

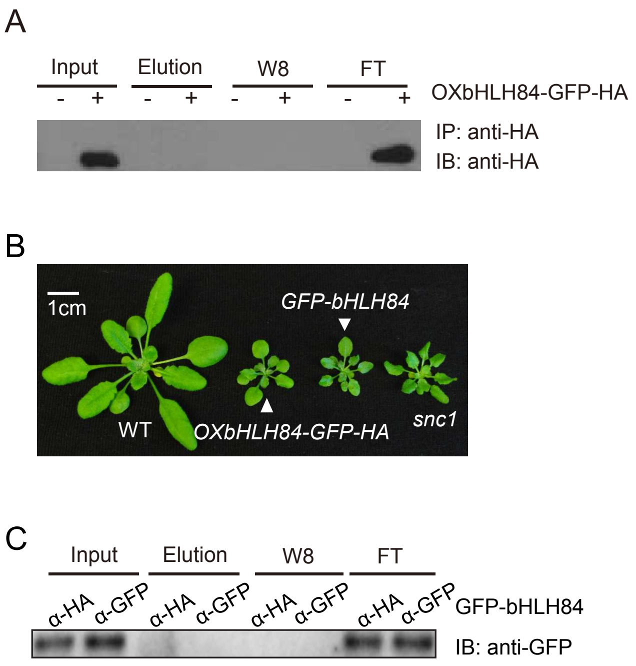

Figure S8. Nuclear immunoprecipitation (IP) of bHLH84 with C-terminal HA tag or N-terminal GFP tag in Arabidopsis.

A. Nuclear IP of OXbHLH84-GFP-HA in Arabidopsis. The nuclei of *OXbHLH84-GFP-HA* transgenic plants were isolated and nuclear proteins were extracted and subjected to IP with anti-HA beads, using a previously described method [63]. Input indicates protein sample before IP. Elution indicates eluted protein sample. W8 indicates the 8th wash sample. FT (Flow Through) indicates the unbound proteins. "-" indicates sample without OXbHLH84-GFP-HA, which served as a negative control, while "+" indicates OXbHLH84-GFP-HA sample. IP: immnoprecipitation. IB: immunoblot.

B. Morphology of four-week-old soil-grown WT, *snc1, OXbHLH84-GFP-HA* and *GFP-bHLH84* epitope-tagged transgenic plants.

C. Nuclear IP of bHLH84 with N-terminus GFP tag in Arabidopsis. Similar IP procedure as Figure S8A was carried out except that the negative control was *GFP-bHLH84* sample incubated with anti-HA beads.
