# Supplementary material for: NLR-Associating Transcription Factor bHLH84 and Its Paralogs Function Redundantly in Plant Immunity
Source: PLoS Pathog. 2014 Aug 21;10(8):e1004312. doi: 10.1371/journal.ppat.1004312 (PMC4140859; doi:10.1371/journal.ppat.1004312)
Supplement: Figure S9 — bHLH84-HA accelerates HR in N. benthamiana caused by SNC1-FLAG. A. Representative leaf HR morphology of N. benthamiana at 36 hours and 40 hours post-infiltration of Agrobacterium. Four-week-old N. benthamiana leaves were co-infiltrated with Agrobacterium containing the indicated constructs at OD600 = 0.2. Agrobacterium containing pCambia1300-35S -FLAG or pCambia1300-35S –HA empty vectors (EV) served as controls. The cycles label the infiltrated regions. The red arrow points to the HR symptom caused by the co-infiltration of Agrobacterium containing pCambia1300-35-SNC1-FLAG and pCambia1300-35S- bHLH84-HA. B. The percentage of N. benthamiana leaves showing HR after infiltration with Agrobacterium containing the indicated constructs at the indicated time point. Six leaves of 3 plants were used for inoculations in a pattern similar to that shown in Figure S9A. At the indicated time point, leaves displaying visible gray and slightly shinny cell death spots were recorded as exhibiting HR symptom. For each treatment, the percentage of HR were calculated using the number of leaves showing HR symptom at the indicated time points divided by the total number of inoculated leaves. More quantitative measurements of ion leakage, which reflects the level of cell death, are presented in Figure 7A. (PDF) [file ppat.1004312.s009.pdf]

Figure S9

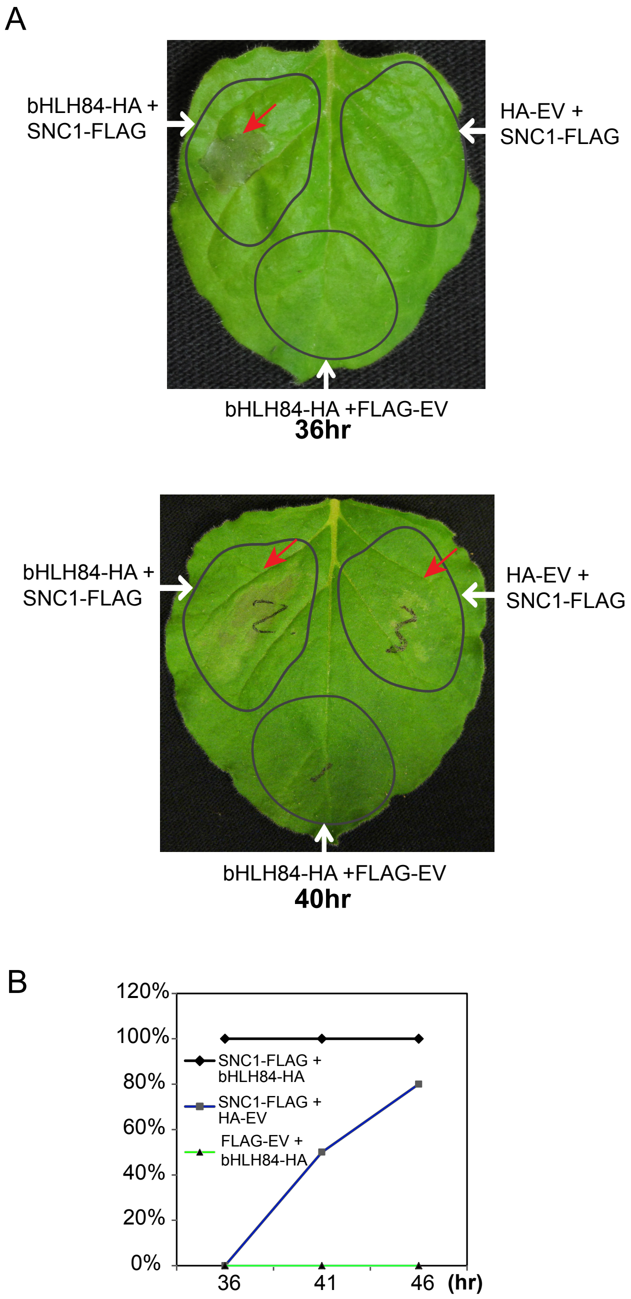

Figure S9. bHLH84-HA accelerates HR in *N. benthamiana* caused by SNC1-FLAG.

A. Representative leaf HR morphology of *N. benthamiana* at 36 hours and 40 hours post-infiltration of *Agrobacterium.* Four-week-old *N. benthamiana* leaves were co-infiltrated with *Agrobacterium* containing the indicated constructs at OD_{600}=0.2. Agrobacterium containing *pCambia1300-35S* -FLAG or *pCambia1300-35S* –HA empty vectors (EV) served as controls. The cycles label the infiltrated regions. The red arrow points to the HR symptom caused by the co-infiltration of *Agrobacterium* containing *pCambia1300-35-SNC1-FLAG* and *pCambia1300-35S*- bHLH84-HA.

B. The percentage of *N. benthamiana* leaves showing HR after infiltration with *Agrobacterium* containing the indicated constructs at the indicated time point. Six leaves of 3 plants were used for inoculations in a pattern similar to that shown in Figure S9A. At the indicated time point, leaves displaying visible gray and slightly shinny cell death spots were recorded as exhibiting HR symptom. For each treatment, the percentage of HR were calculated using the number of leaves showing HR symptom at the indicated time points divided by the total number of inoculated leaves. More quantitative measurements of ion leakage, which reflects the level of cell death, are presented in Figure 7A.
