# Supplementary material for: NLR-Associating Transcription Factor bHLH84 and Its Paralogs Function Redundantly in Plant Immunity
Source: PLoS Pathog. 2014 Aug 21;10(8):e1004312. doi: 10.1371/journal.ppat.1004312 (PMC4140859; doi:10.1371/journal.ppat.1004312)
Supplement: Figure S10 — bHLH84 does not interact with RPS2-FLAG (A) or RPS6-FLAG (B). A. bHLH84-HA could not be pulled down by RPS2-FLAG when co-expressed in N. benthamiana leaves. Four-week-old N. benthamiana plants were co-infiltrated with Agrobacterium containing pCambia1300-35S-bHLH84-HA and pCambia 1300-35S-RPS2-FLAG at OD600 = 0.2 for each strain. Total protein extracted from 6 g of N. benthamiana leaves collected 33 hours post-inoculation was subjected to IP. Half of the sample was incubated with anti-FLAG beads while the other half was incubated with protein A agarose beads without the conjugated anti-FLAG antibody, which serves as negative IP control. Input indicates protein sample before IP. Elution indicates protein sample competitively eluted from the beads by 3×FLAG peptides. B. bHLH84-HA could not be pulled down by RPS6-FLAG when co-expressed in N. benthamiana leaves. Similar procedures were carried out as described in Figure S10A. (PDF) [file ppat.1004312.s010.pdf]

A

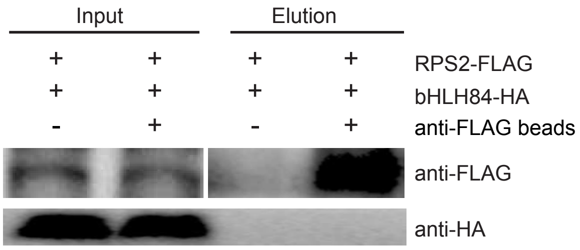

B

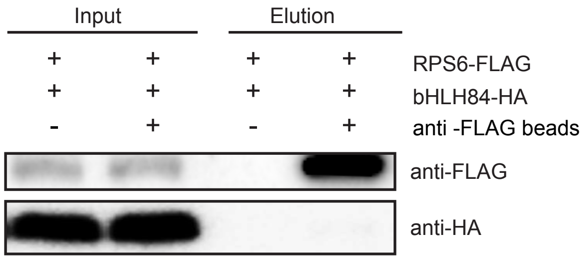

Figure S10. bHLH84 does not interact with RPS2-FLAG (A) or RPS6-FLAG (B).

A. bHLH84-HA could not be pulled down by RPS2-FLAG when co-expressed in *N. benthamiana* leaves. Four-week-old *N. benthamiana* plants were co-infiltrated with *Agrobacterium* containing *pCambia1300-35S-bHLH84-HA* and *pCambia 1300-35S-RPS2-FLAG* at OD_{600}=0.2 for each strain. Total protein extracted from 6 g of *N. benthamiana* leaves collected 33 hours post-inoculation was subjected to IP. Half of the sample was incubated with anti-FLAG beads while the other half was incubated with protein A agarose beads without the conjugated anti-FLAG antibody, which serves as negative IP control. Input indicates protein sample before IP. Elution indicates protein sample competitively eluted from the beads by 3xFLAG peptides.

B. bHLH84-HA could not be pulled down by RPS6-FLAG when co-expressed in *N. benthamiana* leaves. Similar procedures were carried out as described in Figure S10A.
