# Supplementary material for: NLR-Associating Transcription Factor bHLH84 and Its Paralogs Function Redundantly in Plant Immunity
Source: PLoS Pathog. 2014 Aug 21;10(8):e1004312. doi: 10.1371/journal.ppat.1004312 (PMC4140859; doi:10.1371/journal.ppat.1004312)
Supplement: Figure S11 — Both bHLH84-HA and TPR1-HA can be immunoprecipitated by SNC1-FLAG when all three protein were co-expressed in N. benthamiana . Four-week-old N. benthamiana leaves were infiltrated with Agrobacterium strains carrying the indicated constructs. Total protein extracted from 3 g of N. benthamiana leaves for each infiltration collected 36 hours post-inoculation were subjected to IP. Three samples were incubated with anti-FLAG beads while one sample was incubated with protein A agarose beads, which served as negative control. Two western blots of bHLH84-HA from two independent experiments are presented. (PDF) [file ppat.1004312.s011.pdf]

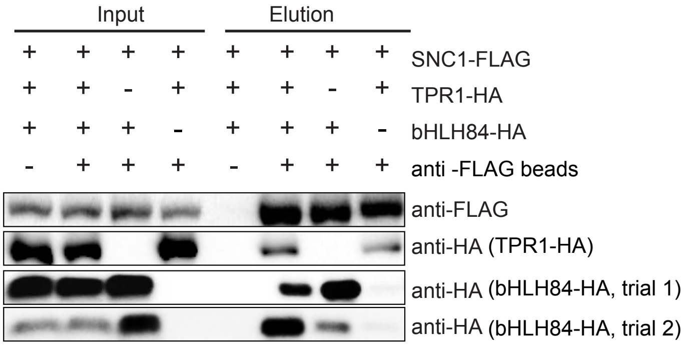

Figure S11. Both bHLH84-HA and TPR1-HA can be immunoprecipitated by SNC1-FLAG when all three protein were co-expressed in *N. benthamiana*.

Four-week-old *N. benthamiana* leaves were infiltrated with *Agrobacterium* strains carrying the indicated constructs. Total protein extracted from 3 g of *N. benthamiana* leaves for each infiltration collected 36 hours post-inoculation were subjected to IP. Three samples were incubated with anti-FLAG beads while one sample was incubated with protein A agarose beads, which served as negative control. Two western blots of bHLH84-HA from two independent experiments are presented.
