# Supplementary material for: NLR-Associating Transcription Factor bHLH84 and Its Paralogs Function Redundantly in Plant Immunity
Source: PLoS Pathog. 2014 Aug 21;10(8):e1004312. doi: 10.1371/journal.ppat.1004312 (PMC4140859; doi:10.1371/journal.ppat.1004312)
Supplement: Figure S12 — bHLH84 does not interact with truncated SNC1 protein in yeast. A. Schematic diagram of constructs containing the indicated domains of SNC1.The full-length protein of SNC1 (SNC1-FL) is encoded by 6 exons as shown in the diagram. B. bHLH84 did not interact with the truncated SNC1 segments in yeast-two-hybrid assays. Yeast strain Y1347 cotransformed with bHLH84 fused with AD in PGADT7 vector and PGBKT7 constructs with indicated truncated SNC1 fused with BD are plated on SD-Leu-Trp and SD-Leu-Trp-His with 3 mM 3AT. BD-EV indicates the empty PGBKT7 vector. Yeast growth on SD-Leu-Trp indicates successful cotransformation of the constructs and serves as inoculum control. (PDF) [file ppat.1004312.s012.pdf]

Figure S12

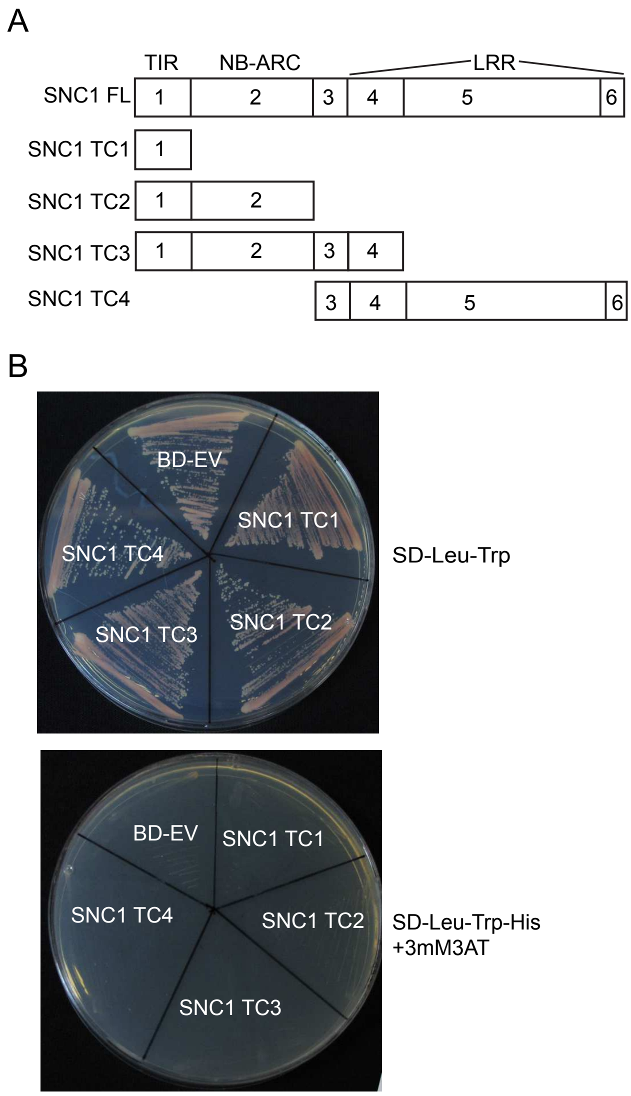

Figure S12. bHLH84 does not interact with truncated SNC1 protein in yeast.

A. Schematic diagram of constructs containing the indicated domains of SNC1.The full-length protein of SNC1 (SNC1-FL) is encoded by 6 exons as shown in the diagram.

B. bHLH84 did not interact with the truncated SNC1 segments in yeast-two-hybrid assays. Yeast strain Y1347 cotransformed with *bHLH84* fused with *AD* in *PGADT7* vector and *PGBKT7* constructs with indicated truncated *SNC1* fused with *BD* are plated on SD-Leu-Trp and SD-Leu-Trp-His with 3mM 3AT. BD-EV indicates the empty PGBKT7 vector. Yeast growth on SD-Leu-Trp indicates successful cotransformation of the constructs and serves as inoculum control.
