# Supplementary material for: NLR-Associating Transcription Factor bHLH84 and Its Paralogs Function Redundantly in Plant Immunity
Source: PLoS Pathog. 2014 Aug 21;10(8):e1004312. doi: 10.1371/journal.ppat.1004312 (PMC4140859; doi:10.1371/journal.ppat.1004312)
Supplement: Figure S13 — bHLH84 does not interact with EDS1 or PAD4 in N. benthamiana . bHLH84-HA could not be pulled down by EDS1-FLAG or PAD4-FLAG when co-expressed in N. benthamiana leaves. Four-week-old N. benthamiana plants were co-infiltrated with Agrobacterium containing pCambia1300-bHLH84-HA and pCambia 1305-EDS1-FLAG or pCambia 1305-PAD4-FLAG at OD600 = 0.2 for each strain. Similar IP procedure was carried out as described in Figure 7D. Protein extracts incubated with protein A agarose beads without anti-FLAG serve as negative control. EDS1-FLAG or PAD4-FLAG are indicated by the arrow. The band in EDS1 elution fraction indicated with an * presumably represents a degradation or cleavage product of EDS1. (PDF) [file ppat.1004312.s013.pdf]

Figure S13

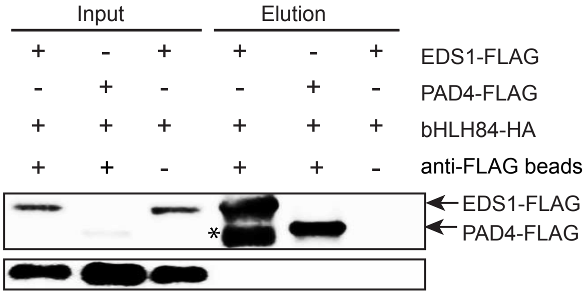

Figure S13. bHLH84 does not interact with EDS1 or PAD4 in *N. benthamiana.*

bHLH84-HA could not be pulled down by EDS1-FLAG or PAD4-FLAG when co-expressed in *N. benthamiana* leaves. Four-week-old *N. benthamiana* plants were co-infiltrated with *Agrobacterium* containing *pCambia1300-bHLH84-HA* and *pCambia 1305-EDS1-FLAG* or *pCambia 1305-PAD4-FLAG* at OD_{600}=0.2 for each strain. Similar IP procedure was carried out as described in Figure 7D. Protein extracts incubated with protein A agarose beads without anti-FLAG serve as negative control. EDS1-FLAG or PAD4-FLAG are indicated by the arrow. The band in EDS1 elution fraction indicated with an * presumably represents a degradation or cleavage product of EDS1.
