# Supplementary material for: NLR-Associating Transcription Factor bHLH84 and Its Paralogs Function Redundantly in Plant Immunity
Source: PLoS Pathog. 2014 Aug 21;10(8):e1004312. doi: 10.1371/journal.ppat.1004312 (PMC4140859; doi:10.1371/journal.ppat.1004312)
Supplement: Figure S14 — A working model on how SNC1 activates plant immunity through interacting with different transcription factors in distinct protein complexes. The bHLH84-SNC1 complex may bind to the promoter regions of downstream positive regulators of plant immunity by recognizing the specific cis-elements to activate defense response. This complex most likely contains other unidentified components. It functions separately from and in parallel with the HDA19-TPRs-SNC1 complex, which represses negative regulators of immunity, such as DND1 and DND2, possibly through an unknown DNA-binding protein. “?”s represent unknown protein partners. (PDF) [file ppat.1004312.s014.pdf]

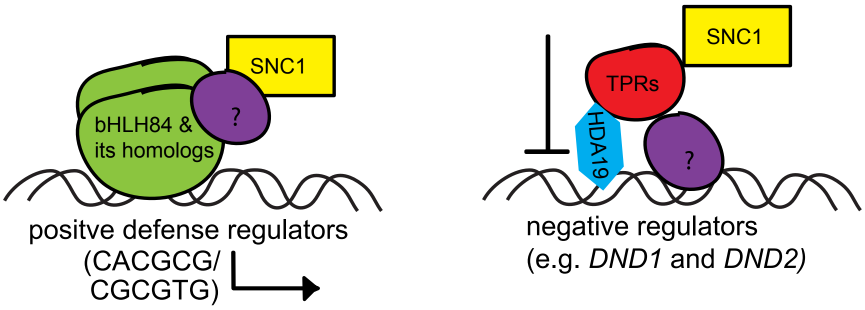

Figure S14. A working model on how SNC1 activates plant immunity through interacting with different transcription factors in distinct protein complexes.

The bHLH84-SNC1 complex may bind to the promoter regions of downstream positive regulators of plant immunity by recognizing the specific cis-elements to activate defense response. This complex most likely contains other unidentified components. It functions separately from and in parallel with the HDA19-TPRs-SNC1 complex, which represses negative regulators of immunity, such as *DND1* and *DND2*, possibly through an unknown DNA-binding protein. “?”s represent unknown protein partners.
