# Supplementary material for: NLR-Associating Transcription Factor bHLH84 and Its Paralogs Function Redundantly in Plant Immunity
Source: PLoS Pathog. 2014 Aug 21;10(8):e1004312. doi: 10.1371/journal.ppat.1004312 (PMC4140859; doi:10.1371/journal.ppat.1004312)
Supplement: Figure S15 — Immunoblot detection of bHLH84-GFP-HA in ChIP (Chromatin Immunoprecipitation) samples. bHLH84-GFP-HA and snc1 seedlings (10 g of tissue for each genotype) were cross-linked with 1% formaldehyde. Nuclei were extracted, sonicated, and centrifuged to obtain supernatant which was diluted in the ChIP IP buffer and subjected to anti-HA precipitation. Input indicates protein sample before IP. Elution indicates eluted protein sample. “−” indicates sample without OXbHLH84-GFP-HA, which serves as negative control. “+” indicates OXbHLH84-GFP-HA sample. The ChIP experiment was carried out as previously described [17]. (PDF) [file ppat.1004312.s015.pdf]

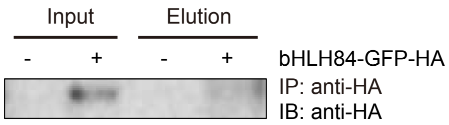

Figure S15. Immunoblot detection of bHLH84-GFP-HA in ChIP (Chromatin Immunoprecipitation) samples.

bHLH84-GFP-HA and *snc1* seedlings (10g of tissue for each genotype) were cross-linked with 1% formaldehyde. Nuclei were extracted, sonicated, and centrifuged to obtain supernatant which was diluted in the ChIP IP buffer and subjected to anti-HA precipitation. Input indicates protein sample before IP. Elution indicates eluted protein sample. “-” indicates sample without OXbHLH84-GFP-HA, which serves as negative control. “+” indicates OXbHLH84-GFP-HA sample. The ChIP experiment was carried out as previously described [17].
