# Supplementary material for: NLR-Associating Transcription Factor bHLH84 and Its Paralogs Function Redundantly in Plant Immunity
Source: PLoS Pathog. 2014 Aug 21;10(8):e1004312. doi: 10.1371/journal.ppat.1004312 (PMC4140859; doi:10.1371/journal.ppat.1004312)
Supplement: Figure S16 — bHLH84 exhibits DNA binding activity with N1- and N2-box cis elements. A. The sequences of the tested DNA fragments in the yeast-one-hybrid assays. The tripled cis-element sequences are indicated with the underline. B. bHLH84 only exhibited detectable binding activity to N1- and N2-boxes. Yeast cell Y187 cotransformed with constructs harboring bHLH84 fused with GAL4 activation domain (AD) and pHIS2 constructs containing the indicated DNA fragment were plated on the SD-Leu-Trp and SD-Leu-Trp-His with 60 mM or 100 mM 3-Amino-1,2,4-Triazole (3AT). The growth of yeast cells on SD-Leu-Trp-His with 60 mM 3AT or 100 mM 3AT reflects the binding activity of bHLH84 while that on SD-Leu-Trp serves as loading control. Yeast cells cotransformed with pHIS2 empty vector (EV) and bHLH84-AD together with yeast strains cotransformed with AD empty vector (EV) and pHIS2 constructs with the indicated DNA fragments served as negative controls. (PDF) [file ppat.1004312.s016.pdf]

Figure S16

A

N1-box: GGTCACGCGGCCACGCGGACACGCGAG

N2-box: GGTCGCGTGGCCGCGTGGACGCGTGAG

N3-Box: GGTCACGAGGCCACGAGGACACGAGAG

G-box: GGTCACGTGGCCACGTGGACACGTGAG

B

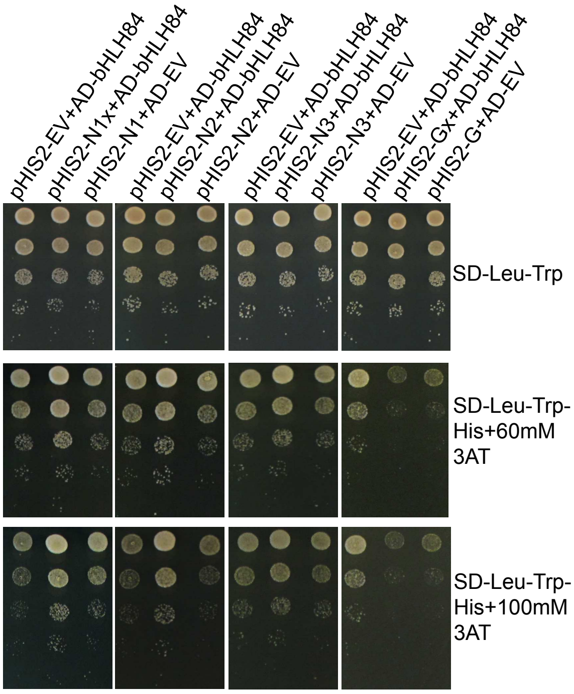

Figure S16. bHLH84 exhibts DNA binding activity with N1- and N2-box cis elements.

A. The sequences of the tested DNA fragments in the yeast-one-hybrid assays. The tripled cis-element sequences are indicated with the underline.

B. bHLH84 only exhibited detectable binding activity to N1- and N2-boxes. Yeast cell Y187 cotransfromed with constructs harboring *bHLH84* fused with *GAL4* activation domain (AD) and *pHIS2* constructs containing the indicated DNA fragment were plated on the SD-Leu-Trp and SD-Leu-Trp-His with 60 mM or 100mM 3-Amino-1,2,4-Triazole (3AT). The growth of yeast cells on SD-Leu-Trp-His with 60mM 3AT or 100mM 3AT reflects the binding activity of bHLH84 while that on SD-Leu-Trp serves as loading control. Yeast cells cotransfromed with *pHIS2* empty vector (EV and *bHLH84-AD* together with yeast strains cotransformed with *AD* empty vector (EV) and *pHIS2* constructs with the indicated DNA fragments served as negative controls.
