# Supplementary material for: NLR-Associating Transcription Factor bHLH84 and Its Paralogs Function Redundantly in Plant Immunity
Source: PLoS Pathog. 2014 Aug 21;10(8):e1004312. doi: 10.1371/journal.ppat.1004312 (PMC4140859; doi:10.1371/journal.ppat.1004312)
Supplement: Table S1 — Summary of the transcription factors screened and their overexpression phenotypes in snc1 and Col-0 background. In snc1 background, “−” indicates a suppressing phenotype, and “+” indicates an enhancing phenotype. In Col-0, “+”indicates dwarfism caused by the transgene. “NC” indicates no change. The number of “−” and “+” is correlated with the degrees of the phenotype. Note: A few genes, such as At2g18670, At5g10380 and At5g67340, have been recently annotated as RING proteins or U-box proteins. They are more likely to be E3 ligases rather than TFs. (PDF) [file ppat.1004312.s017.pdf]

Gene ID	Transcription factor type	*snc1*	Col-0
At1g06160	ERF/AP2	NC	NC
At1g08930	Zinc finger	-	NC
At1g13260	AP2/B3	-	NC
At1g21910	DREB	NC	NC
At1g26800	Zinc finger	NC	NC
At1g28040	Zinc finger	+	NC
At1g62300	WRKY6	NC	NC
At1g67970	HSFA8	NC	NC
At1g68360	Zinc finger	+	NC
At1g68840	ERF2	NC	NC
At1g77570	HSF	NC	NC
At2g14760	bHLH	+	+++
At2g18670	Zinc finger	NC	NC
At2g31230	ERF/AP2	+	+
At2g32460	MYB	NC	NC
At2g47270	bHLH	NC	NC
At3g11020	DREB2B	+	NC
At3g24500	ERF	NC	NC
At3g28210	Zinc finger	NC	NC
At3g45260	Zinc finger	-	NC
At3g46640	MYB	NC	+
At3g50260	DREB	NC	NC
At3g55980	Zinc finger	-	NC
At3g61630	AP2/ERF	NC	NC
At4g01720	WRKY	NC	NC
At4g17500	ERF	NC	NC
At4g27410	NAC	NC	NC
At5g10380	Zinc finger	NC	NC
At5g47220	ERF	NC	NC
At5g49450	bZIP	NC	NC
At5g49700	AT-hook DNA-binding	-	NC
At5g52830	WRKY	-	NC
At5g58620	Zinc finger	-	NC
At5g61590	ERF	+	++
At5g67340	Zinc finger	NC	NC
At5g67450	Zinc finger	NC	NC

Table S1. Summary of the transcription factors screened and their overexpression phenotypes in *snc1* and Col-0 background.

In *snc1* background, “-” indicates a suppressing phenotype, and “+” indicates an enhancing phenotype. In Col-0, “+”indicates dwarfism caused by the transgene. “NC” indicates no change. The number of “-” and “+” is correlated with the degrees of the phenotype. Note: A few genes, such as *At2g18670*, *At5g10380* and *At5g67340*, have been recently annotated as RING proteins or U-box proteins. They are more likely to be E3 ligases rather than TFs.
